# Genetic and Pre- and Postharvest Factors Influencing the Content of Antioxidants in Cucurbit Crops

**DOI:** 10.3390/antiox10060894

**Published:** 2021-06-02

**Authors:** Cecilia Martínez, Juan Luis Valenzuela, Manuel Jamilena

**Affiliations:** Department of Biology and Geology, Agrifood Campus of International Excellence (CeiA3) and CIAIMBITAL Reseach Center, University of Almería, 04120 Almería, Spain; cmartinez@ual.es (C.M.); jvalenzu@ual.es (J.L.V.)

**Keywords:** antioxidants, Cucurbitaceae, genotype, stress, biotechnology, natural variability

## Abstract

Cucurbitaceae is one of the most economically important plant families, and includes some worldwide cultivated species like cucumber, melons, and squashes, and some regionally cultivated and feral species that contribute to the human diet. For centuries, cucurbits have been appreciated because of their nutritional value and, in traditional medicine, because of their ability to alleviate certain ailments. Several studies have demonstrated the remarkable contents of valuable compounds in cucurbits, including antioxidants such as polyphenols, flavonoids, and carotenoids, but also tannins and terpenoids, which are abundant. This antioxidant power is beneficial for human health, but also in facing plant diseases and abiotic stresses. This review brings together data on the antioxidant properties of cucurbit species, addressing the genetic and pre- and postharvest factors that regulate the antioxidant content in different plant organs. Environmental conditions, management, storage, and pre- and postharvest treatments influencing the biosynthesis and activity of antioxidants, together with the biodiversity of this family, are determinant in improving the antioxidant potential of this group of species. Plant breeding, as well as the development of innovative biotechnological approaches, is also leading to new possibilities for exploiting cucurbits as functional products.

## 1. Introduction

The simplest definition of an antioxidant is as a compound that is effective at preventing oxidation. One of the characteristics of oxidation is that it is a chemical reaction that releases cell-damaging free radicals; i.e., reactive and unstable atoms, ions, and molecules with unpaired electrons. Due to their high reactivity, they attack other substances in order to stabilise themselves [1,2]. Although the initial attack results in the pairing of the electron, another free radical is formed. This new free radical works in the same way with a third molecule, continuing the generation of more and more unstable products, setting off a chain reaction. This chain reaction ends when either the free radical is stabilised by an antioxidant, or the reaction chain is stopped by producing a harmless compound [3]. In order to mitigate the risk of cell damage, living organisms present mechanisms that are based on nonenzymatic and enzymatic antioxidant systems that include, among others, different vitamins, phenolic compounds, carotenoid pigments, superoxide dismutases, catalases, peroxidases, and glutathion reductases [4].

When plants have to cope with stressful environmental changes, high production of reactive oxygen species (ROS) occurs in different physiological processes, and therefore in different cellular locations and different organelles. These reactive oxygen species are the main cell-damaging free radicals and include superoxide anions, hydrogen peroxide, hydroxyl radicals, nitric oxide, and peroxynitrite. The induced production of ROS results in the peroxidation of membrane lipids and cell membrane damage [5,6]. The reactions between antioxidant compounds and free radicals are not only dependent on the concentration of both reagents, but also on different factors such as the chemical structure of the molecules involved and the medium and conditions where the reaction takes place. For these reasons, it happens quite often that a compound can show antioxidant activity in vitro but not in vivo. Among other reasons, some free radicals are very short-lived (in the order of nanoseconds), and there is not enough time for the antioxidant compounds to be in the right place at the right time, just when oxidative damage occurs [7].

Antioxidation can take two forms: prevention and chain-breaking. Prevention is primarily based on the scavenging of the initiating radicals in the oxidation chain, while chain-breaking relies on the antioxidant stopping the cascade of reactions by stabilising any free radicals that form in the different links of the oxidation chain. The enzymatic antioxidant system works when the enzymes reduce the energy of free radicals or give up some of their electrons for stabilisation. In addition, they can interrupt the oxidation chain. Superoxide dismutase (SOD), glutathione peroxidase (GPX), catalase (CAT), and glutathione reductase (GR) work together to form a first line of defence. First, SOD suppresses the O_2_^−^ radical by adding an electron to form H_2_O_2_, then the hydrogen peroxide is reduced by CAT and GPX, resulting in H_2_O and oxidised glutathione, and finally the oxidised glutathione is reduced by GR [8]. Three families of SODs are present in plants depending on the metal used as a cofactor: Cu–Zn SODs, Fe SODs, and Mn SODs. These SODs are distributed in different cellular compartments, whereas Fe SODs are found in the chloroplast, Mn SODs are found in the mitochondrion, and the peroxisome Cu–Zn SODs are found in both the chloroplast and the cytoplasm, although there is also evidence of possible extracellular localisation [9,10,11]. Catalase eliminates H_2_O_2_ in two ways: by using oxygen peroxide to oxidise substrates, or by reducing the cellular content of peroxide. It is an enzyme containing a heme group, and is extremely important for removing the hydrogen peroxide produced in the beta-oxidation of fatty acids, in the glyoxylate cycle, and in the catabolism of purines. In various isoenzymes, it can be found in the peroxisome, cytoplasm, and mitochondria [8,12]. Glutathione peroxidase is a multiple isozyme family that reduces H_2_O_2_ or organic hydroperoxides, using glutathione as an electron donor [13,14]. Glutathione reductase is an enzyme that reduces glutathione disulphide, a glutathione using NADPH as an electron donor. Glutathione reductase is predominantly located in chloroplasts, but also is found in mitochondria, peroxisomes, and the cytoplasm [15].

The nonenzymatic antioxidant system usually works by neutralizing free radicals through electron donation. In this process, antioxidants are converted into free radicals but with much less reactivity, because they are neutralised by other nonenzymatic antioxidants. The list of nonenzymatic antioxidants is long: ascorbic acid, carotenoid compounds, selenium, manganese, zinc, flavonoids, etc. Ascorbic acid is a water-soluble ketolactone whose antioxidant mechanism is to transfer a hydrogen atom to the peroxyl radical, inactivate the singlet oxygen, and finally scavenge oxygen [16,17]. The number of carotenoid compounds identified is very high, reaching more than 700. They are formed by a conjugated polyunsaturated chain where the essential free radical inhibition ability of carotenoids lies. The lipophilic character of carotenoids by the polyunsaturated chain enables them to play an important role in protecting cell membranes against free radicals. Besides the stabilization of free radicals by single-electron transfer and hydrogen atoms by reaction with different reductants, it also scavenges radicals through the formation of a carotenoid–radical adduct, when the free radical is added to the polyunsaturated chain [18,19,20]. Phenolic compounds are a diverse group of more than 8000 compounds with an important role in the human and animal diet, as well as in plant defence. Phenolic compounds contain a hydroxyl group that is essential for their antioxidant activity, as they reduce the free radical by transferring a hydrogen atom from this hydroxyl group. Phenolic compounds also base their antioxidant capacity on the transfer of a single electron (SET); the free radical is stabilised, as it has no unpaired electrons. The phenolic compound, on the contrary, is left with an odd number of electrons and distributes the charge throughout their aromatic ring by resonance to remain stable [21,22].

The biosynthesis and activity of antioxidants are affected by multiple factors, including environmental conditions and management, as well as storage conditions and pre- and postharvest treatments [23]. In addition, the high diversity of this family, and the variability of modern cultivars, facilitates the improvement of antioxidant content and activity. In general, the factors that affect the quality of fruits and vegetables can be grouped into two main categories: genotype and environment. The genetic constitution of the plant is the main factor, but there are other extrinsic factors, occurring before, during, and after harvesting, that can also influence the final quality of the product [24,25]. The nutritional status, climatic conditions, crop system, irrigation regime, cultural practices, crop management during harvest and postharvest, storage conditions, and others are key factors that influence the antioxidant profile and content [26]. For example, while high rates of nitrogen fertilisers lower antioxidant content, water shortage results in higher antioxidant content. In this sense, it has been observed that different effects of organic and conventional crop systems lead to increases or decreases in certain antioxidants.

This review aims to provide an updated overview of factors influencing antioxidant content in cucurbit crops, highlighting the genetic, as well as pre- and postharvest, factors enhancing antioxidant properties of cucurbits. We also show the advances in detecting QTL and genes regulating the content of some antioxidants. The review concludes by highlighting several future perspectives for enhancing the quality of cucurbits.

## 2. Variation of Antioxidant Content among Species of the Cucurbitaceae Family

Cucurbitaceae is an extensive family that comprises about 800 species in 130 genera [27]. Edible plants are found in genera *Benincasa*, *Cucurbita*, *Cucumis*, *Citrullus*, *Lagenaria*, *Momordica*, and *Sechium* [28,29], and some of them are the most cultivated crops worldwide (e.g., watermelon, melon, cucumber, and squashes and pumpkins). Among these genera, there are species whose organs exhibit an important accumulation of bioactive compounds with antioxidant activity, such as carotenoids, polyphenols, cucurbitacins, or citrulline, that have a free-radical scavenging ability [30,31].

The study of antioxidant content among cultivars of *Citrullus lanatus* (Thunb.) (Matsun. Et Nakai), *Cucumis melo* (L.), and *Cucurbita* spp., among others, indicates that one of the most relevant factors responsible for the variation is the genotype [32,33,34], but antioxidant content can also vary due to different factors like growing conditions and the ripening stage of the organ.

### 2.1. Cucurbita

The *Cucurbita* genus is composed of five major crop species: *Cucurbita pepo* (L.), *Cucurbita moschata* (Duchesne), *Cucurbita maxima* (Duchesne), *Cucurbita argyrosperma* (Huber), and *Cucurbita ficifolia* (Bouche), as well as other minor crops of regional relevance [27,28]. Fruits, leaves, shoots, and seeds are rich sources of nutrients that offer several benefits for human health [35,36,37,38,39,40], but multiple species of the genus have been also recognized as sources of antioxidants, and are used in traditional medicine [41]. Their fruits are rich in many biologically active compounds, among them carotenoids (α- and β-carotene, β-cryptoxanthin, lutein, and zeaxanthin) and phytosterols [42,43]. The analysis of the total carotenoid content in the flesh of mature fruits in *C. moschata* pointed to maximum values of 148.8 μg/g dry weight DW in ethanol/hexane solvent [44], and 813 μg/g D.W. according to Durante et al. [45] in conventional solvent extraction (CSE). The predominant carotenoids are β-carotene and α-carotene, with amounts of 61.6 μg/g DW and 29.3 μg/g DW, respectively. The β-carotene and α-carotene contents varied among cultivars [46], although variation also was due to the extraction method and the fruit maturation stage. The same is true for mature fruits of *C. maxima*, where carotenoid content was found to vary between 63.7 mg/100 g (acetone) and 132.2 mg/100 g (ethanol/olive oil) DW in a mix of cultivars and using different extraction methods [47,48]. In a different report, the total carotenoid content of 12 pumpkin cultivars (*C. maxima* and *C. pepo*) was found to range from 5 to 160 mg/100 g DW [49]. Moreover, comparison of the antioxidant ability of 18 cultivars of *C. maxima*, *C. moschata*, *C. pepo*, and *C. ficifolia* revealed high intraspecies variability in the genus. The fruits of the Japanese *C. moschata* ‘Kogigu’ had a high quantity of phenolic compounds (70.8 mg/100 g fresh weight, FW). Another popular cultivar, ‘Hokkaido’, exhibited the highest antioxidant and antiradical capacities. The cultivars of *C. maxima* with the highest total polyphenol content, 50.4 and 41.6 chlorogenic equivalents (CAE) mg/100 g FW, respectively, were ‘Indomatrone’ and ‘Bambino’. In addition, ‘Indomatrone’ fruits contained one of the highest levels of salicylic acid (2.56 mg/100 g FW). In *C. pepo,* variation was observed in the phenolic contents, while ‘KamoKamo’ (51.5 CAE/100 g FW) and ‘Sweet Dumpling’ (48.1 CAE/100 g FW) had the highest; the lowest was found in ‘Miranda’ (13.2 CAE/100 g FW). The ‘Angel Hair’ of *C. ficifolia* showed a total phenols content of 20.6 CAE/100 g FW [34].

Besides the fruit flesh, fruit peel and seeds are also good sources of antioxidants such as total polyphenols, total flavonoids, and β-carotene [38,50,51,52]. In fact, pumpkin oil shows antioxidant activity due to the content of squalene, a linear triterpenoid that plays an important role in maintaining oxidation stability [53,54]. The antioxidant activities of a triterpene isolated from *C. argyrosperma* seeds ameliorated oxidative stress, favouring cell protection through different mechanisms, including preventing lipid peroxidation and scavenging oxygen free radicals in vitro [55]. Similarly, *C. ficifolia* antioxidant activity led to the reduction of lipid peroxidation in vivo [56]. Compounds with valuable antioxidant activity were also found in *Cucurbita foetidissima* Kunth, a species in which catechol (a phenolic component) is present in the root, leaves, and stem in concentrations between 0.8 and 1.9 g/100 g DW [57].

### 2.2. Citrullus

Besides the worldwide-consumed watermelon, *Citrullus lanatus*, in the genus *Citrullus,* there are three additional species consumed regionally: the egusi watermelon (*Citrullus mucosospermus* Fursa), the citron watermelon (*Citrullus amarus* Schrad.), and the colocynth (*Citrullus colocynthis* L.) [58]. *C. lanatus* is cultivated for its fruit flesh, while *C. mucosospermus* is cultivated for its fruit flesh and seeds [59]. *C*. *amarus* is cultivated for its edible but hard fruit flesh, which is often cooked or pickled [60]. *C. colocynthis*, also known as bitter apple, is cultivated for its numerous medicinal properties and the oil of its seeds [61].

The nutritional value and antioxidant potential have been studied in different species, cultivars, and organs by using different methods of analysis. Focusing on *C. lanatus*, the fruit extract displays a very high concentration of valuable compounds such as alkaloids, phenols, anthraquinone, tannins, and saponin [62], but especially lycopene and β-carotene, which were measured to be as high as 4537.83 and 308.71 μg/100 g DW, respectively [63]. Traditionally, the carotenoid profile was thought to vary among cultivars of different colours, and the study of 12 cultivars of red, pink, orange, and yellow flesh confirmed this difference, but also highlighted the variation among cultivars with the same colour flesh [33]. Pink-fleshed watermelons are richer in lycopene (>12 µg/g FW), followed by red (≈5 µg/g FW), while lycopene content is under 2 µg/g FW in orange and yellow-fleshed watermelons. Variability of β-carotene (from 4.8 to 47.8 µg/g FW) is found in pink- and red-fleshed cultivars independently of a similar colour, while orange and yellow watermelons have several distinctive carotenoid profiles [33]. These outcomes suggest that orange and yellow watermelons are defective in the initial common carotenoid biosynthesis steps and have mutations in genes involved in the production of intermediate compounds of lycopene biosynthesis [33]. However, Jin et al. [64] reported that the β-carotene content of the desert watermelon (red and pink) was between 6.5 and 134 mg/kg DW, and Liu et al. [65] found 860.50 μg/g DW of β-carotene content in the orange-/yellow-fleshed watermelon ‘Ju-Bao’. In contrast, the β-carotene content reported for *C. amarus* accession PI 255,137 with yellow flesh was 0.1 µg/g FW [33].

Variation was also found for lycopene and total carotenoid content of red-fleshed fruits of seeded and seedless cultivars [66]. The study included 50 commercial cultivars, among which seeded (2n) cultivars varied in lycopene and total carotenoid contents. The minimum value was found in cultivar ‘Black Diamond’ (lycopene = 35.2 mg/kg and total carotenoids = 37.1 mg/kg), and the maximum was observed in the cultivar ‘Olé’, (lycopene = 76.1 mg/kg and total carotenoids = 87.3 mg/kg). In triploid cultivars, the lycopene and mean total carotenoid accumulation was higher. The minimum values of each component were found in ‘Afternoon Delight’, in which lycopene was 60.9 mg/kg and total carotenoids 68.1 mg/kg, while the maximum accumulation was found in ‘Xite’, with 112.4 mg/kg of lycopene and 121.9 mg/kg FW of total carotenoids [66]. Variation in antioxidant content of watermelon fruits, including polyphenols, flavonoids, and lycopene, was also reported by other authors [38,67,68].

Watermelon is rich in citrulline, a nonessential amino acid with hydroxyl radical scavenger ability [69,70]. Citrulline content has been assessed in watermelons, revealing significant variation among cultivars. A comparison of cultivars by flesh colour and seed production showed that yellow watermelons have the highest value (28.5 mg/g DW), while orange- and red-fleshed watermelons showed intermediate and the lowest citrulline content (14.2 mg/g DW and 7.4 mg/g DW, respectively) [69]. The variation also seems to be influenced by seed content. Seeded watermelons had an average of 1.8 mg/g FW of citrulline, while seedless ones showed an average of 2.4 mg/g FW [69,71,72].

Besides the fruit flesh, antioxidant compounds have been found in other organs of *Citrullus* spp. In watermelon seeds, Emmanuel et al. [73] found that flavonoid content was in the range of 0.015 to 0.347 mg CE/g FW, while Adetutu et al. [63] reported 0.05 mg quercetin equivalent (QE)/g FW of flavonoids. Seeds of *C. colocynthis* are also a source of valuable compounds, and the content of antioxidant varies among cultivars [35,74]. As much as 298.88 mg GAE/g (mg gallic acid equivalent per g extract DW) of polyphenols and 219.18 mg GAE/g DW of flavonoids has been extracted from seeds in this species [31]. Roots and leaves have also been reported to be rich in bioactive compounds in other plant species [75]. In leaves, the accumulation of phenolic compounds increases during the maturation of the plant, so they possess higher total flavonoid contents than other plant organs. In *C. colocynthis,* leaves showed values reaching 580 mg/100 g DW of quercetin in ethanol extract, while flowers or roots showed much lower values, barely 1.33 and 7.05 mg/100 g DW, respectively [76]. The root content of total phenol and total flavonoids was found to be 6.35 GAE and 2.52 mg CE/g DW, respectively, whereas the leaf content was 18.6 GAE and 13.9 CE mg/g DW of total phenols and total flavonoids, respectively [76].

### 2.3. Cucumis

The comparison of the antioxidant content of different cucurbits, including pumpkin (*C. maxima*), ash gourd (*Benincasa hispida* Thunb.), watermelon (*C. lanatus*), and melon (*C. melo*), indicates that melon fruit extracts (peel and pulp) show the highest antioxidant activity, which correlates with higher polyphenolic and flavonoid contents [38]. In this beneficial food, there is also variability in antioxidant profiles and amounts among cultivars. The study of the bioactive molecule content in fruits of 10 cultivars under different systems of cultivation and environmental conditions indicated that the cultivar is a major factor determining the antioxidant properties of melons [77]. Moreover, an evaluation by Ganji et al. [78] of the total phenolic content in the peel of the fruit of 12 cultivars showed variation from 0.69 mg GAE/g extract in the ‘Tuscan’ cultivar to 2.96 mg GAE/g extract in the peel of Galia-type ones, cultivated in organic conditions. These results were correlated with the antioxidant activity, 0.26 mg ascorbic acid equivalent/mL extract in Galia and 0.13 mg ascorbic acid equivalent/mL extract in ‘Tuscan’, thus indicating that the Galia melon could be a natural source of health-promoting compounds [78].

Cantaloupe, another important melon consumed as a dessert fruit, also possesses high antioxidant properties [79,80]. The antioxidant content under methanolic extraction of plant organs varied from 1.68 to 26.40 mg GAE/g extract, in ascending order: flesh < seed < skin < stem < leaf. These results are in accordance with what was observed in other plant species, suggesting that the leaf might be the organ with the highest phenolic content in many plants [80,81,82]. The total flavonoid content of cantaloupe, measured as micrograms of rutin equivalents (RUE) per gram of extract, varied considerably from 1.62 to 69.70 µg RUE/g dry extract (methanolic) in the seeds and leaf, respectively [80]. Vella et al. [83] also reported high flavonoid content and antioxidant properties of melon peel (12.27 mg of ascorbic acid equivalent/g of dry extract) and seed (0.31 mg ascorbic acid equivalent/g of dry extract) in cantaloupe melon types.

*Cucumis sativus* (L.) (cucumber) is a worldwide crop cultivated for its fruit. The measure of total phenolic contents, flavonols, and proanthocyanidins in cucumber extract was found to be 9.05 CE, 2.06 QE, and 55.66 mg/100 g FW, respectively [84]. The study of the antioxidant content in different cucumber plant organs, including flesh, peel, and seeds, showed a high content of total flavonoids in the peel (14.02 mg quercetin equivalents/g in ethanolic extract DW (mg QE/g)), while the flesh (0.27 mg QE/g DW, ethanolic) and seeds (0.32 QE/g DW, water) had a much lower content. Total phenols were also higher in the peel (23.08 mg GAE/g DW, ethanolic), followed by the seed (17.59 mg GAE/g DW, water) and the flesh (10.02 mg GAE/g DW, water) [85].

Antioxidant potential has also been measured in *Cucumis africanus* (L). The fruits, leaves, or roots of this plant are used as an emetic, purgative, or enema in the treatment of various ailments. Total phenol, flavonoid, and proanthocyanidin content varies among organs, with higher content of antioxidants in fruits, followed by leaves and roots [86].

### 2.4. Momordica

The fruit pulp, seeds, leaves, and whole plant of the bitter melon (*Momordica charantia* L.) have been studied for their hypoglycemic effects [87]. The content of lycopene has been found to be variable in fruits among *Momordica cochinchinensis* (Spreng.), with a maximum of 2100 μg/g and a minimum of 408 μg/g FW [88,89,90,91]. Researchers have pointed out that the variation between reported values could be due to different species or genotypes and different fruit maturation stages [92]. In fact, the analysis of leaves of indigenous *Momordica dioica* (Roxb.), cultivated *M. charantia*, and indigenous *M. charantia* var. *muricata* showed differences, and revealed the important antioxidant power of the wild species. Alkaloid, flavonoids, sterols, and anthraquinones were the main active compounds in *M. charantia* leaves [36]. The contents of total phenol content ranged between 33.9 and 49.31 tannic acid equivalent/g of extract (TAE/g), and total flavonoids varied from 72.83 mg RUE/g extract in *M. charantia* to 94.16 mg of RUE/g extract in *M. dioica*. Finally, tannins were found to be higher in *M. charantia* var. *muricata* (19.25 TAE/g of extract), followed by *M. dioica* (14.9 TAE/g of extract) and *M. charantia* (8.51 TAE/g of extract) [36] (Table 1). These results reveal marked differences among wild and cultivated species of *Momordica*, which is in accordance with the results found in strawberries, raspberries, and blackberries [93], and suggest an important role of domestication in the loss of valuable compounds in these species. Similarly, the comparison of antioxidant content in the fruits of two cultivars of *M. charantia,* var. *minima* and var. *maxima*, confirmed the variability due to the genotype. The total phenolic content varied from 1.47 to 27.23 mg GAE/100 g DW. The analysis of fruits without seeds and pith led to higher values of ascorbic acid in both cultivars [94]. The variation in total phenolic contents was higher in the whole fruit of var. *maxima* (27.23 mg GAE/100 g DW) and the whole fruit of the var. *minima* (14.82 mg GAE/100 g DW), suggesting that the accumulation is prominent in the seeds and pith [94] (Table 1). Other reports show high variation compared to what was reported by Choo et al. [94], suggesting a strong influence of the genotype and the external factors [95,96,97,98,99,100,101].

### 2.5. Lagenaria

*Lagenaria siceraria* (Mol.)*,* commonly called the bottle gourd or Lauki, is the most popular vegetable of the genus. Due to its curative properties, it has been utilized for the treatment of various diseases [98]. In this species, the leaves, fruits, and seeds are edible and used in the treatment of different ailments. The leaf extract has shown free-radical scavenging activity [99]. Different works showed a high content in leaves of total phenols (195.15 TAE/g extract, ethanolic), flavonoids (76.62 CE/g extract, ethanolic), and tannins (225.98 CE/g extract, acetone), as well as cucurbitacins B and D, and traces of cucurbitacin E [98,101]. The fruit contains triterpenoids; cucurbitacins B, D, G, H; and 22-deoxy cucurbitacin; as well as flavone-C glycosides, with maximum values of phenols and tannins of 111.18 TAE/g extract (acetone) and 47.14 CE/g extract (aqueous), respectively [98,101]. The separate analysis of the fruit epicarp and mesocarp showed that the epicarp is a good source of phenolics [102]. Other studies pointed to different amounts of phenolics in *L. siceraria*, ranging from 11.9 TAE to 233.4 ± 3.0 mg TAE/g extract, suggesting the importance of not only the solvent and the organ used for the extraction, but also the genotype [103,104,105] (Table 1). Finally, lagenin, a ribosome-inactivating protein (RIP) with medicinal properties, has been isolated from the seeds of *L. siceraria* [100].

### 2.6. Other Genera

*Luffa acutangula* (L.) and *Luffa cylindrica* (L.) are consumed because of the medicinal properties of their fruits [106,107]. The total phenolic and flavonoid contents in fruits of *L. acutangula* has been measured under methanol extraction as 24.6 mg GAE/g DW and 43.2 mg QE/g DW, respectively [108]. The analysis of peel extract also shows a remarkable content of bioactive compounds, with high levels of multiple polyphenols [107]. The measurement of the total amounts of antioxidant compounds in dried gourds of *Luffa cylindrica* was about 1%, suggesting that gourds can be a source of antioxidant constituents in the human diet [106].

*Sechium edule* (Jacq.), or chayote, is a pre-Columbian cultivated plant consumed for its fruits. In addition to the fruits, the stems, leaves, and tuberous part of the roots are also eaten. An assessment of the antioxidant compounds indicated that the highest content of flavonoids is found in the leaves (35.0 mg/10 g DW), followed by the roots (30.5 mg/10 g DW), and finally the stems (19.3 mg/10 g DW). Not only the quantity, but also the quality varied among the organs of the plant, indicating that they could be useful for different applications [109].

**Table 1:** Antioxidant compound range content in cucurbit species.

Genus	Species	Organs	Antioxidant Compounds	Concentration ^a^	References
*Cucurbita*	*C. moschata*, *C. maxima*, *C. pepo*, *C.ficifolia*	Skin, flesh	Total carotenoids	5 to 160 mg/100 g	[44,46,47,48,49]
	*C. moschata*, *C. máxima*, *C.pepo*		Total phenolics compounds	13.2 to 70.8 mg CAE/100 g	[34]
	*C. foetidissima*	Roots, leaves, stem	Cathecol	0.8 to 1.9 g/100 g (expressed as pyrocathecol)	[57]
*Citrullus*	*C. lanatus*	Fruit	Lycopene	35.20–76.10 μg/g	[33,63,64,65]
			b-carotene	6.5 to 860 μg/g	
			Total carotenoids	68.1 to 112 μg/g	[66]
			Total phenolic compounds	80.9 to 147.3 μg GAE/g	[67]
				870–910 μg/g	[68]
			Flavonoids	111.3 to 176.1 μg RUE/g	[66]
		Peel	Total phenolic compounds	48.63 mg GAE/100 g	[38]
			Total flavonoid	13.65 mg QE/100 g	
		Fruit	Citrulline	1.18 to 28.5 g/Kg	[69,71,72]
		Oil seeds	Flavonoids	0.015 to 0.347 mg CE/g	[63]
		Fruit		0.05 mg QE/g	
	*C. colocynthis*	Seeds	Polyphenols	298.8 mg/g as gallic acid	[35]
			Flavonoids	219.18 mg/g as catechin	
		Roots	Total phenols	6.35 mg GAE/g	[76]
			Flavonoids	2.52 mg CE/g	
		Leaves	Total phenols	18.6 mg GAE/g	
			Flavonoids	13.9 mg CE/g	
	*C. amarus*	Fruits	b-carotene	0.1 µg/g	[33]
*Cucumis*	*C. melo*	Peel	Total phenolic compounds	0.1 to 296 mg GAE/g	[76]
			Ascorbic acid	0.13 to 0.26 mg/mL extract	
			Chlorogenic acid	0.16 to 1.3 mg/mL extract	
		Seeds	Total flavonoids (as rutin equivalent)	1.62 mg/g	[79]
		Leaves		to 69.70 mg/g	
		Peel	Tannins	309 µg/g	
		Seed		146 µg/g	
	*C. africanus*	Fruit	Total phenols content	44.98 mg/g	[86]
		Leaves		42.31 mg/g	
		Root		20 mg/g	
		Fruit	Proanthocyanidin	504 mg/g	
		Leaves		275.57 mg/g	
		Root		154.93 mg/g	
	*C. sativus*	Fruit	Total phenolic content	9.05 mg/100 g	[84]
			Proanthocyanidin	2.06 mg/100 g	
			Flavonols	55.66 mg/100 g	
		Peel	Total flavonoids	14.2 mg/g	[85]
			Total phenols	23.08 mg GAE/g	
		Flesh	Total flavonoids	0.27 mg QE/g	
			Total phenols	10.02 mg GAE/g	
		Seeds	Total flavonoids	0.32 mg QE/g	
			Total phenols	17.59 GAE	
*Luffa*	*L. acutangula*	Fruit	Total phenolic compounds	24.6 mg/g	[109] [108]
			Total flavonoids	43.2 mg/g	
		Peel	Catechin	22.80 mg/100 g dw	[108]
			Quercetin	21.72 mg/100 g dw	
			Vanillin	19.60 mg/100 g dw	
*Lagenaria*	*L. siceraria*	Leaves	Total phenolic compounds	195.15 mg TAE/g	[102] [99]
			Total flavonoids	76.02 mg CE/g	
			Total tannins	225.98 mg CE/g	
		Fruit	Total phenols	111.18 mg TAE/g	
			Total tannins	47.14 mg CE/g	
		Fruit	Total phenolic compounds	11.9 to 233.4 mg TAE/g	[104,105,106]
*Momordica*	*M. charantia*	Leaves	Total phenols	33.9 to 49.31 TAE/g	[36]
			Tannins	8.51 to 19.25 TAE/g	
			Flavonoids	72.83 RUE/g	
	*M. dioica*		Tannins	14.9 TAE/g	
			Flavonoids	94.16 TAE/g	
	*M. charantia*	Fruit	Ascorbic acid	3.8 to 85 mg/100 g	[96,97,98,99,100]
			Total phenolics compounds	14.82 to 27.23 mg GAE/100 g	[36]
	*M. cochinchinensis*	Fruit	Lycopene	408 to 2073 µg/g	[89,90,91]
*Sechium*	*S. edule*	Leaves	Flavonoids	35.0 mg/10 g	[110]
		Roots		30.5 mg/10 g	
		Stem		19.3 mg/10 g	

^a^ Maximum values found among extraction methods are shown. CAE: chlorogenic acid equivalents; GAE: gallic acid equivalents; QE: quercetin equivalents; CE: catechin equivalents; TAE: tannic acid equivalents; RUE: rutin equivalents.

## 3. Preharvest Factors Affecting Antioxidant Potential of Fruits and Vegetables

The genotype is the most important factor controlling the content and activity of antioxidants in fruits and vegetables, but there are also other preharvest determinants, including environmental conditions, biotic and abiotic stresses, management and cultural practices, and the ripening stage of the edible organ at harvest.

### 3.1. Environmental Conditions

Climatic factors such as temperature, light, relative humidity, etc. influence the organoleptic quality and the antioxidant content of plant organs. In this respect, phenolic compounds are most affected by climatic factors [111,112]. Light seems to have no influence on the carotenoid and ascorbic acid content, but plants and fruits have a higher phenolic content when exposed to higher light intensities, which has been observed in grapes and apples exposed to sunlight [110,113]. In addition to light intensity, light quality significantly influences the content of phenolic compounds, especially anthocyanins. Thus, when lettuce is grown under a high level of ultraviolet radiation, it enhances the content of flavonoids as protective agents [114,115]. Light quality also affects the content of bioactive compounds such as carotenoids and phenols in carrot. According to Martinez-Zamora et al. [116], when carrot sprouts were treated with red, far-red, and blue light, the phenol content increased by more than 45% and the carotenoid content by more than 220% compared to sprouts grown in the dark. In cucumbers, the spectral composition of light affects sensitivity to ultraviolet B radiation. Supplementary UV-B radiation negatively affected the growth of cucumber plants under blue, green, and white light, but did not affect plant growth under red light. Thus, UV-B and red light act synergistically in priming the antioxidant capacity, and thus reduce the negative effects of photoinhibition [117]. A recent paper by Mastropasqua et al. [118] has shown the influence of light on pumpkin sprouts. White, red, and blue light treatments with an irradiance of 110 µmol m^−2^ s^−1^ preserved the contents of ascorbic acid, carotenoids, and anthocyanins, with the authors emphasising that the use of a specific wavelength was able to increase the content of these antioxidants. Light stress was also found to have an important effect on the citrulline content in leaves of *C. melo* and *Citrullus* species [119].

Although cucurbits are considered photophilic plants and are suitable for cultivation in warm, bright locations, it is possible to find a degree of diversity in their tolerance to light and heat stress. *C. moschata* is more adapted to warm conditions and is mostly cultivated in tropical and subtropical regions, whereas *C. maxima* is cultivated in warm, temperate environments [120]. Under stressful conditions, either due to light or heat, oxidative stress is generated, with which the plants cope by producing antioxidant compounds. An interesting correlation was found between the production of antioxidant compounds and susceptibility to heat stress. In *C. moschata*, *C. maxima*, and a hybrid of these two species, Ara et al. [121] found a differential response of antioxidant enzymes to heat stress, being more intense in *C. maxima* (considered heat-sensitive) and less intense in *C. moschata* (considered heat-tolerant). The response was intermediate in the hybrid (considered moderately heat-tolerant). Antioxidant enzymes, such as SOD, showed increased activity in the more heat-tolerant pumpkin.

### 3.2. Drought and Salinity Stress

Drought and salinity stress have a common basis; the plants have to face a very low water potential and overcome an oxidative stress [122].

Under drought stress, cucurbitacin content was found to be higher, implicating these compounds in drought tolerance. Cucurbitacins are a large family of highly oxidized tetracyclic triterpenoids known for their numerous pharmacological effects, including antioxidant and free-radical scavenging properties. There is not much information on the relationship between the different cucurbitacins and drought [123]. Cucurbitacins E and I have been found in different cultivars of *Lagenaria siceraria,* and it is believed that they could be involved in plant drought response [124]. Water shortage causes oxidative stress to plant cells. Fan et al. [125], who exposed cucumber seedlings to different degrees of water stress by treating them with different concentrations of polyethylene glycol, found that drought stress increased malondialdehyde content, as well as hydrogen peroxide, while antioxidant enzymes like SOD, CAT, and glutathione reductase showed lower activity. These authors pointed out that an enhanced water stress breaks the balance between antioxidant activities and the generation of ROS [125].

Various approaches have been proposed in an attempt to alleviate the adverse effects of water stress. One such approach is the application of nitric oxide, which stimulates the production of antioxidants. Hamurcu et al. [126] carried out a study using two watermelon cultivars, one of them drought-tolerant, the ‘KAR98’ cultivar, and another cultivar considered less tolerant, the ‘REDE’ cultivar. Both were subjected to water stress by adding polyethylene glycol to the culture medium, and the results were compared with unstressed control and with a treatment with nitric oxide provided by a donor, sodium nitroprusside (SNP). As expected, under stressed conditions plants not only showed lower growth rates, but also enhanced concentration of MDA and a differential response of antioxidant enzymes. These authors concluded that the effect of NO application depends on the type of stress, the species studied, and the genotype [126]. Fan et al. and Hamurcu et al. [125,126] have also reported that the stress response in terms of antioxidant synthesis is dependent on the degree of stress and the type of stress applied.

Salt stress is one of the most important abiotic stresses and one of the most economically damaging to agriculture. When plants are subjected to this type of stress, their productivity decreases significantly because, on the one hand, plants have difficulty capturing water, and on the other hand, they suffer from sodium toxicity. These two factors lead to the production of reactive oxygen species, and plants have to cope with this oxidative stress [127]. When plants have a higher content of antioxidant compounds, either naturally or induced, they can cope with the oxidative stress produced by salt. Thus, antioxidant enzyme activities such as CAT, POD, APOX, and others are increased in plants under stress, and their activities are correlated with stress tolerance [128]. The range of tolerance to salt stress in cucurbits is high, with species and even cultivars having different degrees of tolerance. A study of three cucumber cultivars found differences in response to salt stress. The most tolerant cultivar had a higher osmotic adjustment capacity and a higher activity of antioxidant enzymes such as SOD, CAT, and APOX, as well as a lower degree of membrane damage, as evidenced by lower MDA content [129]. In general, in cucurbits, as in other plants, salt sensitivity leads to a lower activity of antioxidant enzymes such as CAT and APX, although for other enzymes such as SOD, this is not so clear [130,131,132]. MDA content is a clear indicator of the damage to cell membranes caused by stress. In response to salt stress, MDA content has been found to vary between species and between genotypes. Although some studies report that different genotypes of squash and melon increase the MDA content with salt treatments, some authors suggest that the relationship between salt stress and increased MDA content depends on the genotype, with some melon genotypes showing lower MDA content in response to salt treatments [133].

In conclusion, plants respond to environmental stresses by producing a variable amount of antioxidant compounds of nutraceutical value. For this reason, it has been postulated that abiotic stress can be a tool to improve the nutraceutical quality of fruits and vegetables [134]. However, for the application of abiotic stress to become a useful tool, it is essential that its implementation does not affect crop yields, so knowledge of the mechanisms that plants use to counteract stressful environments is essential. As Toscano et al. [134] pointed out, the use of controlled abiotic stress is a new frontier in agricultural science that can lead to products of higher nutraceutical quality.

### 3.3. Preharvest Treatments

Preharvest treatments consist of the application of various compounds with or without hormonal activity to the plants during the growing season. Among the compounds with hormonal activity, the most common are methyl jasmonic acid and its derivatives [135], as well as salicylic acid [136], compounds with auxinic activity, gibberellic acid, or abscisic acid [137]. Among the compounds without hormonal activity, calcium, fungicides, pesticides, foliar fertilisers, allelochemical compounds, and NO-donor compounds such as SNP stand out [138,139].

The effects of preharvest treatments with jasmonic acid and its derivatives have been studied on different fruits and vegetables, including cucurbits. In most cases, the purpose of these treatments was to enhance disease resistance and fruit size and quality. Its use to increase bioactive compounds in fruits is more limited, and most studies have been carried out on stone and berry fruits [140]. Studies have also been conducted on cucurbits, many of them aimed to improve disease resistance, quality, or resistance to chilling injury during the subsequent postharvest period [141].

Preharvest treatments with salicylic acid (SA) and its derivatives have been used to induce systemic acquired resistance and also to reduce chilling injury during cold storage, although little information is available [142]. In general, preharvest SA treatments increased fruit quality and fruit resistance to fungal pathogens such as *Penicillium* sp., *Monillinia* sp., and others. An increase in the phenol and anthocyanin content and antioxidant activity was also found, mainly based on the increase of antioxidant enzymes such as CAT and POX [136,143]. This same mechanism, based on increasing phenolic compounds and the activity of enzymes such as POX, CAT, and SOD, is the basis for inducing resistance to *Botrytis* in several fruits and vegetables [144]. In cucurbits, preharvest applications of SA have been less studied, but they have been used to improve fruit quality, especially in melons, as well as disease resistance [145]. In melons of the variety ‘Hami’, it was found that foliar treatments of 1 mol/L acetylsalicylic acid applied 14, 21, and 28 days after flowering reduced lesions in fruits inoculated with *Trichothecium roseum*. The treatments also induced a higher content in lignin and flavonoids, and a higher activity of cinnamic acid-4-hydroxylase, PAL, POD, and PPO [146]. SA treatments have also been used to enhance cold tolerance in cucurbits. SA increased the yield of the plants and the marketable quality of the fruits. On the other hand, the effects of foliar application of salicylic acid on nutraceutical quality depended on the concentrations applied. Thus, doses of 0.075 mM AS did not affect nutraceutical quality, while higher doses such as 0.5 mM caused an increase in bioactive compounds such as total flavonoids and total phenols, which significantly enhanced the nutraceutical quality of the fruits [147,148].

The applications of compounds with hormonal activity have been aimed at reducing deterioration or increasing resistance to different stresses or even increasing fruit quality [149]. For example, benzyl-aminopurine treatments delay softening and cell-wall degradation, and were found to improve fruit quality in summer squash [150].

Many other compounds without hormonal activity have been applied exogenously with the intention of improving fruit quality characteristics or inducing resistance to both biotic and abiotic stresses by enhancing the antioxidant system. Some experiments were carried out with cinnamic acid, which is considered a secondary metabolite that induces oxidative stress and stimulates the activity of antioxidant enzymes. The application of cinnamic acid to cucumber seedlings alleviated chilling injury. The increased resistance of seedlings to the cold was based on the increased antioxidant activity of enzymes such as SOD, CAT, GPX, and others such as PX and GSH. This antioxidant activity reduced membrane lipid peroxidation and increased cold tolerance. In conclusion, the application of cinnamic acid induced an antioxidant activity that was revealed by a lower MDA content and ROS levels [151].

### 3.4. Grafting

Horticultural grafting originated in Asia and is now widespread throughout the world in order to obtain improved yields, tolerate soil-borne diseases, and resist environmental stresses such as salinity and drought [152]. Melons and cucumbers are the most widely grafted cultivated cucurbit species. The most frequently used rootstocks for melon and watermelon grafting are interspecific hybrids of *Cucurbita maxima* × *Cucurbita moschata*, but in watermelons the use of *Lagenaria siceraria* as well as wild *Citrullus* species are also used as rootstocks. *Luffa cilindrica*, as well as *Cucumis* and interspecific hybrids of *Cucurbita,* are the most widely used rootstocks in cucumbers [153,154,155].

A higher activity of the antioxidant enzyme system (SOD, CAT, PX, and other enzymes), as well as a lower content of ROS and other oxidative stressors such as H_2_O_2_ or a lower content of MDA, is the result when different species are grafted in order to withstand stresses such as salinity and drought [156]. Cucumber grafted on Luffa withstood water stress better thanks to the induction of enzymes such as SOD, dehydroascorbate dehydrogenase (DHAR), and guaiacol peroxidase (GPOD). In addition, some of this resistance to water stress was also associated with the role of ABA and its signalling [157]. Although some authors point out that grafted cucurbits have a higher content of antioxidant compounds that may be beneficial for the human diet [158], other authors indicate that in watermelons, the content of total phenols was not affected by the scion/rootstock combination. In grafted watermelons, there were higher phenolic concentrations than in ungrafted watermelons, but these differences were not significant if the rootstock was a commercial hybrid of *C. maxima* × *C. moschata* or was *Lagenaria* [159]. In this respect, although the use of different rootstocks does not seem to affect the lycopene content, other factors, such as the growing region, should be taken into account. The interaction between the growing region and the rootstock plays an important role in determining fruit quality, and it is therefore important to evaluate and select the most suitable scion/rootstock combination depending on the growing region, as it significantly influences fruit quality, lycopene and vitamin C content, and antioxidant activity [160].

Cucumber plants grafted on different rootstocks (*Cucurbita moschata, Lagenaria siceraria, Citrullus lanatus*, and *Cucurbita maxima*) were more tolerant to salinity and showed better growth and development than ungrafted plants. Salt tolerance was based on the fact that grafted plants were able to maintain better photosynthetic efficiency and a higher activity of antioxidant enzymes (SOD, POX, CAT, APOX, and GR). It was also observed that resistance to salt stress was correlated with the expression of genes related to stress and photosynthesis [161]. Grafting can help cucumber plants to withstand high temperature stress [162]. Thus, cucumber plants self-grafted and grafted onto *Momordica* did not show significant differences for photosynthetic efficiency if the growing temperature was 28 °C, but at 42 °C, grafted cucumber plants showed higher efficiency than ungrafted and self-grafted plants. Authors suggested that the resistance to heat stress in cucumber seedlings was due to the production of antioxidant compounds, as well as to an improvement in photosynthetic efficiency and a higher production of polyamines [162].

Grafting is also common in melon. In an experiment to test the effects of water stress, cantaloupe melon plants were grafted onto *Cucurbita maxima* and grown under three different water stresses (20, 30, and 40 kPa). At harvest time, the fruits of the grafted plants showed higher firmness, higher vitamin C content, and increased CAT and GPX activity; however, SOD activity was higher in the ungrafted plants. In short, grafting led to better fruit quality [163]. However, in a recent study it was pointed out that the use of grafted melons on nematode-resistant rootstocks is only necessary if the soil is infested with nematodes, as grafting per se does not induce higher fruit yield or fruit quality [164]. So, it is likely that the selection of the rootstocks may be an essential requirement to increase antioxidant content and fruit quality of melon and other grafted crop species.

### 3.5. Farming System

Due to their importance as valuable traditional crops, cucurbit production systems have been deeply studied. Among all the techniques, the fertiliser type and dose applied are the aspect with the stronger impact on antioxidant content. In general terms, when nitrogen fertilisation rates increase, the antioxidant content decreases. Thus, doses of nitrogen over 100 kg/ha in pumpkin led to a reduction of up to 30% in antioxidant content in the fruits [165]. Similar results were found for other cucurbits such as melon [166] or cucumber [167]. On the contrary, plants accumulate a higher content of flavonoids and other bioactive compounds when nitrogen fertilisation is limited, and it was concluded that the application of synthetic fertilisers reduced the antioxidant content when nitrogen is easily available to the plant [165,166,167]. Consequently, organic and low-input farming systems of cucurbit crops, where fertilisers are of natural origin, were able to increase the level of antioxidants in comparison with conventional farming systems. Thus, Kopczyńska et al. [168] found that under organic conditions, zucchini fruits had higher antioxidant contents, specifically phenolic acids. Similarly, melon organic production enhanced fruit antioxidant contents compared with conventional production [77]. The application of digestate as a fertiliser for organic farming in cucumber has proven to increase the content of phenols and flavonoids, but also that of other antioxidant, anti-inflammatory, and antitumoral compounds, including neohesperidine and hesperitin, and others such as naringin and narirutin, which reduce the risk of cardiovascular diseases [169].

## 4. Postharvest Factors

Postharvest storage is a common practice that allows production to be managed according to different needs: transport, distribution, and marketing. In general, cold storage is the most widely used postharvest tool to maintain fruit quality and delay senescence and ripening. The influence of storage on antioxidant content is highly variable, depending on factors as diverse as the type of fruit or organ stored, its state of maturity, the storage time and temperature, etc. [170]. In addition, it must be taken into account that different postharvest technologies will influence the content and evolution of the antioxidant capacity.

### 4.1. Cold Storage

Cold storage can alter the antioxidant content of fruits and vegetables [171]. When storage time is long enough, low temperatures can result in postharvest chilling injury, whereby the content of antioxidant compounds can be also altered. For example, the vitamin C content decreased in cucumbers during storage at 5 °C, but did not change if cucumbers were stored at 20 °C [172]. Cold storage can induce the formation of ROS in many species such as cucumbers, zucchini, melons, and others [173]. This ROS production induces the lipid peroxidation associated with chilling injury, which, in the case of cucumbers, causes peroxidation of plastid membranes [174]. However, an induction of AOX has been found, which is a way to divert electrons in the respiratory chain and thus reduce the generation of ROS [175]. There is a strong correlation between the developmental stage of the fruit and its susceptibility to chilling injury. In cucumbers, the expression of antioxidant enzymes is induced at later stages of development, which makes the fruit more tolerant to chilling injury [176].

### 4.2. Heat Treatments

Heat treatments have been shown to be useful for improving the preservation of fruits and vegetables. These treatments are based on bathing plant products in hot water to induce the synthesis of polyamines and stimulate the antioxidant system, providing additional help to withstand stress during storage [36,113]. Heat treatments can also be applied by hot air, or by preconditioning the fruits prior to cold storage. In one way or another, these heat treatments reduce lipid peroxidation, reduce H_2_O_2_ production, and increase synthesis of antioxidant enzymes. In zucchini and cucumbers, preconditioning is useful for increasing fruit resistance to cold storage, which is manifested by lower MDA and ROS content, as well as a higher synthesis of ROS scavengers and enhanced activity of antioxidant enzymes. The result is a lower incidence of chilling injury [177,178,179,180,181].

### 4.3. Packaging

Packaging is part of the postharvest handling of fruit and vegetables and aims to protect the product from external agents. The types of packaging vary widely, although some of them are highly technical, such as modified atmosphere packaging, active packaging, and individual shrink-wrapping (ISW). Modified atmosphere packaging, as well as the use of edible coatings or ISW, alters the composition of the gases surrounding the fruit; this altered atmospheric composition is associated with lower oxidative stress and higher content of polyamines [182,183]. When the atmospheric modification includes a high level of O_2_, the antioxidant activity of SOD, APX, and CAT is induced, as well as an increase in the content of compounds with antioxidant activity such as phenols. In zucchini, it was found that ISW reduces LOX activity as well as H_2_O_2_ and MDA accumulation, and the fruit of very cold-sensitive cultivars can be stored at very low temperatures [184]. Packaging of minimally processed fresh produce in a modified atmosphere also offers a good opportunity to extend the postharvest life of ready-to-eat products. The combination of this technique with other techniques, such as UV treatment, has been applied to watermelon [185]. In minimally processed watermelon slices, lycopene content was not affected, but the phenolic content was lower, possibly because phenolic compounds could be used as antioxidants against the oxidative damage caused by ultraviolet radiation [185].

### 4.4. Chemical Postharvest Treatment

Numerous compounds are applied after the harvest to increase the shelf life of fruits and vegetables. The compounds are the same as those used in preharvest studies. Methyl jasmonic acid treatments in cucumbers reduced chilling injury when kept at a temperature as low as 5 °C, the main effect being to inhibit H_2_O_2_ accumulation [186]. Melatonin is a compound that has multiple functions in plants, including delaying senescence and exerting an antioxidant effect. It is often used in postharvest trials to extend the shelf life of fruits. The application of melatonin stimulates both the enzymatic and nonenzymatic antioxidant systems, eliminating the production of ROS and thus maintaining the integrity of the cell membranes, which translates into an extended postharvest life. It also induces endogenous production of salicylic acid and jasmonic acid, which increase fruit resistance to pathogens by preventing or reducing spoilage [187]. However, when using melatonin, both the concentration used and the duration of the treatment must be taken into account, as these are two factors that strongly influence the success of the treatments. Different concentrations have been studied in cucumbers; and the best results were found with a 2 h treatment duration and a melatonin concentration of 500 μM [188]. However, the long duration of the treatment can be considered a limitation to practical applicability. Experiments have also been carried out in which melatonin was used in conjunction with other compounds. Thus, the simultaneous use of melatonin and ethanol effectively alleviated the lipid peroxidation of membranes, delaying the deterioration in bitter melon fruits [189]. SA, as well as its derivative, methyl salicylic acid, is responsible for various metabolic and physiological processes in plants. It is considered a safe product with the potential to control postharvest losses. SA intervenes in the plant defence system by inducing the expression of AOX and increasing the synthesis of antioxidant enzymes, as well as inducing the activity of PPO, PAL, and POD during storage. In general, it increases the total antioxidant capacity (TAC) of the fruit, as well as the AOX transcript levels. Treatments with SA and MeSA induce the synthesis of heat-shock proteins (HSPs), which are best known for their involvement in high-temperature stress, but are also useful in other stressful situations. Treatments with SA and MeSA prior to low-temperature storage induced the biosynthesis of HSPs, thus improving the cold resistance of SA-treated fruit [144,190]. Postharvest treatments with SA have been used on the main cucurbits, including cucumber, melons, zucchini, *Momordica,* and luffa [144,148,191,192].

Many other compounds without hormonal activity have been applied to fruits to improve their tolerance to postharvest stresses by enhancing the antioxidant system. Chitosan is widely used as an edible coating to keep produce fresh, mainly through reducing water loss from the fruit. In cucumbers, pitting, the main symptom of chilling injury, is reduced by treatment with chitosan oligosaccharides, concomitantly with an upregulation of APX, GR, SOD and CAT, and HSP70 and HSP45.9 genes [193]. *Aloe vera* gel is also used in postharvest treatments. Its antimicrobial properties are very useful to delay fruit spoilage. When applied to cold-stored fruits and vegetables, they exhibit a lower production of free radicals such as superoxide ions, a lower production of hydrogen peroxide, a higher anthocyanin content, and an increase in the activity of the antioxidant enzymes CAT, SOD, and APX. It is also very useful in delaying browning, as it induces POD [194].

## 5. Genetic Control of Bioactive Compound Production in Cucurbit Species

Vegetables represent an important source of bioactive molecules with antioxidant power. Developing new varieties with enhanced content in polyphenols, as well as other antioxidant compounds, is a major goal for breeding. A large diversity in phenolic acid contents has been found among cultivars and wild relatives of many vegetable crops, including cucurbits. Thus, the identification sources of variation in the accumulation of polyphenols, carotenoids, flavonoids, and other antioxidants, as well as the identification of the genes enhancing their production, is going to be a challenge for breeding in the coming years.

In cucurbits, breeding programs have mostly focused on resistance to biotic stresses, tolerance to abiotic stresses, longer shelf life, and early flowering transition, but also on the colour and flavour of the fruit. The interest of consumers in the potential benefits of fruits and vegetables for improving health leads to a new standard for breeding enhancing contents in bioactive compounds in fruits and seeds. Many bioactive molecules, such as hydrosoluble vitamins, carotenoids, and phenolics, show antioxidant activity by preventing the formation of reactive oxygen species, scavenging free radicals, or repairing or removing damaged molecules [195,196,197]. Developing superior cultivars with antioxidant value requires a detailed knowledge of the genetics control of these traits. The most relevant data on the genetic control of antioxidant contents in the most important cucurbit species are summarized below.

### 5.1. Cucurbita

*Cucurbita* spp. are popular around the world for food and ornamental purposes, with wild, feral, and landrace germplasms; old cultivars; modern cultivars; and hybrids conserved in gene banks around the world [28,198]. Many of these accessions have contributed to improve crop yield and resistance to pathogens, and could support the development of cultivars with high-quality fruit and increased antioxidant accumulation. Breeding for high-quality mature fruit flesh has been done in *C. maxima* ‘Buttercup’ [199], acorn squash (*C. pepo*) [200], and tropical pumpkins (*C. moschata*) [201]. In the *Cucurbita* genus, *C. pepo*, the fruits of which are usually consumed when immature, shows high variability in terms of fruit morphology and external and internal colour. Carotenoids present in the flesh of *Cucurbita* impart yellow and orange coloration, but it varies greatly among *Cucurbita* spp. and accessions [46,202,203,204], and tends to increase during storage [205]. *C. maxima* and *C. moschata* cultivars have been reported to be rich in carotenoids [46,206]. A recent study about ovary coloration, carotenoid content, and fruit flesh colour in *C. maxima* used F6 inbred recombinant lines (RILs) to detect QTLs’ link to these traits. Significant QTLs (LOD > 3) for carotenoid content were detected in chromosomes 2, 4, and 14. QTLs on chromosomes 2 and 4 were the most meaningful, being correlated with α-carotene, β-carotene, lutein, zeaxanthin, antheraxanthin, and violaxanthin contents [207]. Similarly, the analysis of an F2 segregating population of *C. moschata* led to the identification of as many as nine different QTLs, explaining the lutein, α-carotene, β-carotene, and total carotenoid content [208].

In *C. pepo*, fruit colour is under the control of 13 loci [209]. Immature fruits’ colour is green or yellow, also showing variation in terms of flavour and vitamin A content, being higher in zucchini, crookneck, and straightneck morphotypes than in the scallop morphotype [210]. The interaction of the dominant alleles *B* and *L2* confers an intense orange colour to fruit flesh and causes an increase in carotenoid content by as much as 15-fold [211,212]. The selection for these alleles could be of interest to improve antioxidant potential of new cultivars.

### 5.2. Citrullus

Even though the variance in fruit shape, size, colour, flavour, and nutrient composition in cultivated modern watermelons is very high, the genetic base of these varieties is narrow, due to selection targeting yield and desirable fruit qualities [213]. For these reasons, natural variability is a valuable resource to improve the quality and quantity of bioactive compounds, not only in the fruit, but also in other plant organs. Moreover, primitive dessert watermelon (*C. lanatus*) landraces and other species of the genus are consumed for their seeds or cooked flesh; these accessions have a low sugar content and might be a source of bioactive compounds such as citrulline, ascorbic acid, flavonoids, and carotenoids (α-, β-carotene and lycopene) [213].

Different studies of natural variability have demonstrated the potential of primitive watermelon varieties to increase the content of bioactive compounds in modern cultivars. The study of the metabolic profiling of flesh samples of mature fruits across 40 accessions led to the discovery and quantification of 617 distinct metabolic traits, and 300 metabolites were annotated [70], finding high variability in metabolites’ accumulation among the accessions. More importantly, 385 metabolites (62.40%) displayed a broad-sense heritability greater than 0.5, suggesting that their accumulation was under the control of heritable factors [214]. In fact, the study of 414 accessions through a genome-wide association study (GWAS) allowed for the discovery or confirmation of different genomic regions associated with seeds and flesh colour. As many as 13 regions were associated with seed coat colour, with the strongest being located in chromosome 3, overlapping a previously detected QTL (*qrc-c3-1*) and within the position of a *polyphenol oxidase* gene that polymerizes o-quinones to produce black, brown, or red pigments [215,216,217]. Besides seed colour, two signals were found to be associated with flesh colour on chromosomes 2 and 4. QTL at chromosome 2 (*QBRX2-1*) coincides with the position of a *tonoplast sugar transporter* gene (*ClTST2*) that leads to colour development. The position of chromosome 4 overlaps with a previous QTL (*FC4.1*) [218] and with the position of a *lycopene β-cyclase* (*LCYB*), a gene implicated in carotenoid biosynthesis [216]. Furthermore, the analysis of 209 *C. lanatus* accessions showed that red- or pink-fleshed watermelons carry a change in a conserved position of the LCYB protein. Orange and yellow accessions of *C. lanatus*, *C. colocynthis*, *C. amarus*, and *C. mucosospermus* had the wild-type allele [216,219], suggesting that this mutation increases lycopene accumulation. Moreover, a previous study identified a QTL, *qFC.1*, the position of which correlates with the *Phytoene synthase 1* (*PSY1*) locus, which also is implicated in carotenoid synthesis. This gene is highly expressed in flesh during fruit maturation, leading to lycopene accumulation [218,219,220,221,222].

### 5.3. Cucumis

*Cucumis* melon fruits’ flesh colour also depends on the combination of chlorophyll and carotenoid pigments, resulting in white, green, and orange fruit flesh. β-carotene is the most important for orange flesh and colour intensity. The study of colour in this species led to the identification of two major genes that act epistatically: *green flesh* (*Gf/gf*) and *white flesh* (*Wf/wf*). Genotype *gfgf* has either white (*Wf-*) or green flesh (*wfwf*) [223,224,225]. Green flesh (*gf*) was mapped to chromosome 9 [226,227], and white flesh (*wf*) was mapped to chromosome 8 [227]. QTLs for carotene content are colocated with these loci [228]. Recently, the *green flesh* locus has been identified as the *CmOr* gene, which regulates β-carotene accumulation in orange-fleshed fruit, but not at the carotenoids’ biosynthesis level [229]. A single SNP that leads to an arginine-to-histidine change in the CmOr protein leads to β-carotene accumulation in orange-fleshed fruit. Recent studies suggest that the *OR* gene regulates chromoplast biogenesis [230]. Besides these, another QTL of carotenoid content is located to chromosome 6. This QTL has been consistently detected in different mapping populations evaluated in different environments [225,231,232] (Table 2).

More recently, the study through genotyping by sequencing (GBS) of a recombinant inbred line population, developed using two commercial cultivars, ‘Védrantais’ (cantalupensis group) and ‘Piel de Sapo’ (inodorus group), supported the construction of a high-density map and the identification of a QTL for carotenoid content in the flesh of ripe fruit (*CARQU9*.*1*) in linkage group IX (LOD = 16.82) explaining 58% of the variance [233].

The study of β-carotene content and flesh colour in cucumbers using different populations has led to multiple models of genetic control. It has been suggested that two recessive genes control the inheritance of β-carotene accumulation in the mesocarp, while one recessive gene controls its accumulation in the endocarp [234]. Earlier studies analysed the segregation of yellow and orange internal colour, indicating differences in the general combining ability (GCA) at immature and mature stages. The additive genetic effect for carotenoid accumulation at the immature stage and additive and nonadditive factors are important for orange colour expression in mature fruits [212,235]. Moreover, Bo et al. [236] evaluated the cross of ‘XIS’ (Xishuangbanna gourd), with high content in β-carotene and orange flesh, with a cultivated cucumber with no β-carotene. The F1 resulted in a fruit without colour in the mesocarp, indicating that orange colouration (β-carotene) is controlled by a recessive gene. The analysis of 124 F7 RILs and a pool of genetically diverse lines revealed that the character is controlled by a single locus, designated *ore,* linked to a SSR marker (SSR07706) on chromosome 3. On the other hand, the cross of two inbred lines, PI200815 (yellow flesh) and 931 (white flesh), was used to identify a single recessive gene (*yf*) controlling yellow colour formation. The QTL analysis identified a genomic region of 149.0 kb on chromosome 7 with 21 candidate genes [237] (Table 2).

**Table 2:** Identification of QTLs and genes controlling the production of antioxidant compounds in cucurbit species.

Species	Trait/Loci	Linkage Group/Chromosome	Analysis to Identify the Region (Population of Analysis)	Genes within the Región	Reference
*C. maxima*	Carotenoids content (α-carotene, β-carotene, lutein, zeaxanthin, antheraxanthin and violaxanthin)	II, IV, XIV	QTL (RIL)		[207]
*C. moschata*	Lutein, α-carotene, β-carotene and total carotenoids content	II, VIII, XI, XV, XVII, XX	QTL (F2)		[210]
*Citrullus/C. lanatus*	seed coat colour	III	GWAS (414 accessions of *Citrullus* spp.)/QTL (RIL)	29 candidate genes, among them a *polyphenol oxidase*,	[216,217]
*Citrullus*	Flesh colour	II and IV	GWAS (414 accessions of *Citrullus* spp.)	*tonoplast sugar transporter* (*ClTST2*) *lycopene β-cyclase* (*LCYB*)	[217,220]
*C. lanatus*	Flesh colour and β-carotene accumulation	I	QTL (F_2:3_ population)	*Phytoene synthase* (*PSY*)	[222]
*C. melo*	Carotenoid content, flesh colour (*green flesh* and *white flesh* loci)	VIII, IX	QTL (F_2:3_ population and RIL)	*CmOr*	[229,231,234]
*C. melo*	Flesh colour, total carotenoids, β-carotene; phytoene; α-carotene; lutein	I, II, IV, VI, VIII, IX, XII	QTL (RI, F_2_ and DHLs population)		[227,233,234]
*C. sativus*	Flesh colour	VII	QTL (F2)	Locus *yf*: 21 candidates within the region	[238]

### 5.4. Luffa

The genetic control of the antioxidant content of *Luffa acutangula* fruit flesh was analysed by studying 21 F1 hybrids constructed by half diallel crosses. The antioxidant content was predominantly attributed to dominant genes with a low narrow sense of heritability, suggesting a nonadditive type of gene action, and that hybrid breeding is the best approach for the genetic improvement of the trait [239].

## 6. Biotechnological Approaches

### 6.1. Mutants

Mutant collections, developed by physical and chemical treatments, are a suitable way to produce variability of interest for breeding. Usually, this is done using an elite line with a high quality and quantity of valuable traits. Among cucurbit species, it has generated multiple ethyl methane sulfonate (EMS) collections. There are three in *C. sativus* [238,240,241], one in *C. melo* [242,243], one in *C. lanatus* [244], and two in *C. pepo* [245,246]. Of them, the collection by Chen et al. [247] identified a mutant with changes in flesh colour, and the collection by Xue et al. [240] identified two families with alterations in fruit colour. In two of the collections of *C. melo* and one of *C. pepo*, researchers studied changes in colour and the impact of mutations in genes of the carotenoid biosynthesis pathway [243,245]. González et al. [243] analysed mutations in the *CmPDS* (*phytoene desaturase*) gene, involved in carotenoid synthesis, though no phenotype connected with fruit flesh colour was found. In the collection generated by Tadmor et al. [242], one family segregated for a yellow–orange fruit flesh (*yofI*) accumulated prolycopene as the major pigment. The *yofI* phenotype is due to a truncated form of CRTISO (carotenoid isomerase) that isomerizes prolycopene in translycopene, which is cyclized to β-carotene. The early carotenoid biosynthesis genes are upregulated in *yofI* fruits, which are richer in phytoene and f-carotene [248]. The *Cucurbita pepo* collection of Vicente-Dolera et al. [245] was evaluated for two genes of the carotenoid pathway, *LCYb* (*lycopene beta cyclase*) and *PSY* (*phytoene synthase*). Seven and eight missense mutations were found in the *LCYB* and *PSY* genes, respectively, although the authors did not discuss the corresponding phenotypes. Further analyses are needed to find suitable resources for increasing carotenoids and other antioxidant compounds in future breeding programs.

### 6.2. Gene-Transformation and Genome-Editing Approaches

The generation of interesting traits in plants can be achieved by either transgenesis or genome-editing techniques, including clustered regularly interspaced short palindromic repeat (CRISPR) and associated protein9 (Cas9). While the first leads to the insertion of foreign genes in plant genomes, the second allows the introduction of precise mutations or substitutions within one plant gene [247]. Both lead to specific genetic changes. CRISPR/Cas9 genome editing has been used to generate knockout mutations in a *phytoene desaturase* gene of *C. lanatus* (*ClPDS*). The mutated lines had pure or mosaic albino phenotypes [249]. In *C. lanatus*, the knockdown of the *ClPHT4;2* gene in red-fleshed accession 97,103 by RNA interference led to a decrease in carotenoid accumulation in fruit flesh. The expression of *ClPHT4*;2, a chromoplast-localized phosphate transporter, is dramatically upregulated during flesh carotenoid accumulation and the acquisition of red colour. The suppression of the gene by RNAi technology decreased carotenoid accumulation in conjunction with a diluted red colour of the flesh and reduced *ClPHT4;2* transcription abundance [250]. *ClPSY*, *ClPDS*, and *ClCHYb* (*beta-carotene hydroxylase*) genes in the carotenoid biosynthesis pathway were also downregulated.

In cucumbers, the *phytoene synthase-2a carotene desaturase* (*PAC*) gene has been successfully introduced in cucumber using *Agrobacterium*-mediated cotyledonary node transformation [251]. The results of RT-PCR and Northern blot demonstrated that the transgenic *PAC* gene can be used to develop β-carotene transgenic crops in *Cucurbitaceae* [251].

### 6.3. Tissue Culture

In vitro culture of plant tissue is a tool to increase the efficacy and speed of breeding. From the point of view of antioxidant value, it allows us to improve the ability of the plant to generate more bioactive compounds of better quality. Tissue culture can raise a whole plant from a segment of a mother plant in a medium containing nutrients and phytohormones, favouring plant development. In this way, it is possible to enhance the production of secondary metabolites [252]. Secondary metabolites that are produced in a plant in response to exogenous stimuli include many interesting molecules such as terpenoids, steroids, alkaloids, nonribosomal polypeptides, enzyme cofactors, fatty-acid-derived substances, and nitrogen-containing compounds. They are a source of natural colours, flavours, and bioactive compounds [253].

In cucurbits, many works have demonstrated the valuable production of bioactive compounds in tissue culture. In *C. anguria*, *M. charantia*, and *M. dioica*, the production of phenolic compounds in transgenic and nontransgenic roots was demonstrated to be efficient, with the production being higher in transgenic versions of the three species [254,255,256,257]. In vitro cultures with jasmonic and salicilyc acids enhanced polyphenol production in *M. charantia* [258]. On the other hand, the addition of sucrose, cytokinin, and GA3 to a Murashige and Skoog (MS) medium resulted in an increase in flavonol induction due to shoot proliferation in *C. anguria* [37].

Tissue culture of cucurbits has been demonstrated to be useful in the production of other bioactive compounds. *Momordica charantia* is a natural producer of charantin, which has antidiabetic properties. Though the production of charantin is higher in the leaves and fruit than in the hairy roots, the culture shows some advantages in relation to the simplification of the extraction of charantin [258]. The cucurbit *Gynostemma pentaphyllum* produced several saponin glycosides, the chemical group to which the triterpenoid saponins belong. Cucurbitacins are triterpenoids responsible for the bitter taste in cucurbits like cucumbers, melons, squashes, pumpkins, and the watermelon family [259]. The commercial sources of *G. pentaphyllum* are highly productive in gypenosides. Hairy root cultures showed less production of gypenosides, but transformed roots showed some advantages: they could synthesize significant quantities of secondary metabolites, with a growing time shorter compared with field plants, and the production was independent of seasonal and geographical climate changes [259,260].

Other interesting types of compounds found in cucurbits are the ribosome-inactivating proteins (RIPs). RIPs are toxic N-glycosidases that depurinate eukaryotic and prokaryotic rRNAs, thereby arresting protein synthesis during translation. RIPs are either enzymatically active single polypeptides (type I) or heterodimers (type II). Some RIPs (type I) have antifungal and antibacterial activities [261,262]. Different authors have evaluated the RIP activity of extracts from different parts and organs of *L. cylindrica* growing in vitro in a MS medium, and found inhibitory activity [263,264,265]. The production of RIPs from transgenic cultures of *L. cylindrica* established with *Agrobacterium rhizogenes* strain 1855 was shown to inhibit the growth of in vitro human melanoma cell lines, suggesting that RIPs can be produced and purified from hairy root cultures [265]. Transgenic hairy root lines of *Trichosanthes kirilowii* were also found to produce valuable RIPs [266].

## 7. Conclusions and Future Perspectives

Vegetables are a source of many beneficial antioxidant compounds that vary in quality and quantity among species. Cucurbitaceae is an extensive family of plants that includes important crops consumed for their fruits. This organ accumulates a high amount of antioxidant compounds in species cultivated worldwide like *C. moschata*, *C. maxima*, *C. pepo*, *C. lanatus*, *C. sativus*, and *C. melo*. Some other minor crops of regional importance such as *C. colocynthis*, *M. charantia*, and *L. siceraria*, as well as other wild or feral species such as *Sechium* spp. or *M. dioica*, also show a remarkable content of antioxidants not only in the fruits, but also in the leaves, roots, and other organs. The antioxidant properties of important cucurbit crop species have been analysed in several studies [267,268,269,270,271], but the potential of many others remains unexplored. The high variability of the family, which includes more than 800 species in about 130 genera [27], might lead us to find new sources of bioactive compounds among unexplored species, but also to identify new functional molecules.

Polyphenols and flavonoids, together with other antioxidants like carotenoids, and terpenoids like cucurbitacin or citrulline, are commonly found in organs of cucurbit species. Polyphenols (gallic, chlorogenic, caffeic, vanillic, p-coumaric acid, etc.) and flavonoids (catechin, myricetin, and quercetin), as well as ascorbic acid, are known as potential sources of antioxidants in cucurbits [34,35,63,76,80,98,271,272], while carotenoids, which contribute to colour and flavour, play an important role in maintaining good health through their antioxidant activity and because of their contribution of vitamin A to the diet [271,273]. Finally, cucurbits are also an important source of cucurbitacins, a tetracyclic triterpenoid that shows a wide spectrum of biological activities such as cytotoxic, hepatoprotective, cardiovascular, antidiabetic, antioxidant, anti-inflammatory, analgesic, and antiproliferative [274].

The latest research is also favouring the discovery of genes and alleles that improve the biosynthesis pathways of antioxidant compounds. The wealth of available genomic information on cucurbit species is improving the detection of new QTLs and genes regulating the biosynthesis of antioxidant compounds. All these data will not only improve our knowledge of the genetic regulation of antioxidant production, but also the establishment of biotechnological approaches that will facilitate the enhanced production of antioxidants in cucurbit species.

## References

[1] Bandyopadhyay U., Das D., Banerjee R.K. (1999). Reactive oxygen species: Oxidative damage and pathogenesis. Curr. Sci..

[2] Atta E.M., Mohamed N.H., Abdelgawad A.A. (2017). Antioxidants: An overview on the natural and synthetic types. Eur. Chem. Bull..

[3] Brewer M.S. (2011). Natural Antioxidants: Sources, Compounds, Mechanisms of Action, and Potential Applications. Compr. Rev. Food Sci. Food Saf..

[4] Salehi B., Martorell M., Arbiser J., Sureda A., Martins N., Maurya P., Sharifi-Rad M., Kumar P., Sharifi-Rad J. (2018). Antioxidants: Positive or Negative Actors?. Biomolecules.

[5] Del Río L.A., López-Huertas E. (2016). ROS Generation in Peroxisomes and its Role in Cell Signaling. Plant Cell Physiol..

[6] Valenzuela J.L., Manzano S., Palma F., Carvajal F., Garrido D., Jamilena M. (2017). Oxidative stress associated with chilling injury in immature fruit: Postharvest technological and biotechnological solutions. Int. J. Mol. Sci..

[7] Santos Sánchez N., Salas-Coronado R., Villanueva C., Hernandez-Carlos B. (2019). Antioxidant Compounds and Their Antioxidant Mechanism.

[8] Pandhair V., Sekhon B.S. (2006). Reactive oxygen species and antioxidants in plants: An overview. J. Plant Biochem. Biotechnol..

[9] Tyagi S., Singh S.P., Upadhyay S.K., Singh S., Upadhyay S., Pandey A., Kumar S. (2019). Role of Superoxide Dismutases (SODs) in Stress Tolerance in Plants. Molecular Approaches in Plant Biology and Environmental Challenges. Energy, Environment, and Sustainability.

[10] Alscher R.G., Erturk N., Heath L.S. (2002). Role of superoxide dismutases (SODs) in controlling oxidative stress in plants. J. Exp. Bot..

[11] del Rio L.A., Sandalio L.M., Altomare D.A., Zilinskas B.A. (2003). Mitochondrial and peroxisomal manganese superoxide dismutase: Differential expression during leaf senescence. J. Exp. Bot..

[12] Palma J.M., Mateos R.M., López-Jaramillo J., Rodríguez-Ruiz M., González-Gordo S., Lechuga-Sancho A.M., Corpas F.J. (2020). Plant catalases as NO and H_2_S targets. Redox Biol..

[13] Navrot N., Collin V., Gualberto J., Gelhaye E., Hirasawa M., Rey P., Knaff D.B., Issakidis E., Jacquot J.P., Rouhier N. (2006). Plant glutathione peroxidases are functional peroxiredoxins distributed in several subcellular compartments and regulated during biotic and abiotic stresses. Plant Physiol..

[14] Chen M., Li K., Li H., Song C.P., Miao Y. (2017). The glutathione peroxidase gene family in gossypium hirsutum: Genome-wide identification, classification, gene expression and functional analysis. Sci. Rep..

[15] Harshavardhan V.T., Wu T.-M., Hong C.-Y., Hossain M.A., Mostofa M.G., Diaz-Vivancos P., Burritt D.J., Fujita M., Tran L.-S.P. (2017). Glutathione Reductase and Abiotic Stress Tolerance in Plants. Glutathione in Plant Growth, Development, and Stress Tolerance.

[16] Sisein E.A. (2014). Biochemistry of free fadicals and antioxidants. Sch. Acad. J. Biosci..

[17] Bilska K., Wojciechowska N., Alipour S., Kalemba E.M. (2019). Ascorbic acid—The little-known antioxidant in woody plants. Antioxidants.

[18] Sandmann G. (2019). Antioxidant Protection from UV- and Light-Stress Related to Carotenoid Structures. Antioxidants.

[19] Krinsky N.I., Yeum K.J. (2003). Carotenoid-radical interactions. Biochem. Biophys. Res. Commun..

[20] Pérez-Gálvez A., Viera I., Roca M. (2020). Carotenoids and Chlorophylls as Antioxidants. Antioxidants.

[21] Zeb A. (2020). Concept, mechanism, and applications of phenolic antioxidants in foods. J. Food Biochem..

[22] Leopoldini M., Russo N., Toscano M. (2011). The molecular basis of working mechanism of natural polyphenolic antioxidants. Food Chem..

[23] Ramjan M., Ansari M.T. (2018). Factors affecting of fruits, vegetables and its quality. J. Med. Plants.

[24] Tyagi S., Sahay S., Imran M., Rashmi K., Mahesh S.S. (2017). Pre-harvest factors influencing the postharvest quality of fruits: A review. Curr. J. Appl. Sci. Technol..

[25] Scalzo J., Politi A., Pellegrini N., Mezzetti B., Battino M. (2005). Plant genotype affects total antioxidant capacity and phenolic contents in fruit. Nutrition.

[26] Arah I.K., Amaglo H., Kumah E.K., Ofori H. (2015). Preharvest and postharvest factors affecting the quality and shelf life of harvested tomatoes: A mini review. Int. J. Agron..

[27] Esteras C., Nuez F., Picó B., Wang Y.H., Behera T.K., Kole C. (2012). Genetic Diversity Studies in Cucurbits Using Molecular Tools. Genetics, Genomics and Breeding of Cucurbits.

[28] Lebeda A., Widrlechner M.P., Staub J., Ezura H., Zalapa J., Kristkova E., Singh R. (2007). Cucurbits (Cucurbitaceae; *Cucumis* spp., *Cucurbita* spp., *Citrullus* spp.). Genetic Resources, Chromosome Engineering, and Crop Improvement.

[29] Rolnik A., Olas B. (2020). Vegetables from the Cucurbitaceae family and their products: Positive effect on human health. Nutrition.

[30] Rajasree R.S., Francis F., William H. (2016). Phytochemicals of Cucurbitaceae Family—A Review. Int. J. Pharmacogn. Phytochem. Res..

[31] Kaushik U., Aeri V., Mir S.R. (2015). Cucurbitacins—An insight into medicinal leads from nature. Pharmacogn. Rev..

[32] Salehi B., Capanoglu E., Adrar N., Catalkaya G., Shaheen S., Jaffer M., Giri L., Suyal R., Jugran A.K., Calina D. (2019). Cucurbits Plants: A Key Emphasis to Its Pharmacological Potential. Molecules.

[33] Yoo K.S., Bang H., Lee E.J., Crosby K., Patil B.S. (2012). Variation of carotenoid, sugar, and ascorbic acid concentrations in watermelon genotypes and genetic analysis. Hortic. Environ. Biotechnol..

[34] Kostecka-Gugała A., Kruczek M., Ledwożyw-Smoleń I., Kaszycki P. (2020). Antioxidants and health-beneficial nutrients in fruits of eighteen cucurbita cultivars: Analysis of diversity and dietary implications. Molecules.

[35] Benariba N., Djaziri R., Bellakhdar W., Belkacem N., Kadiata M., Malaisse W.J., Sener A. (2013). Phytochemical screening and free radical scavenging activity of *Citrullus colocynthis* seeds extracts. Asian Pac. J. Trop. Biomed..

[36] Nagarani G., Abirami A., Siddhuraju P. (2014). A comparative study on antioxidant potentials, inhibitory activities against key enzymes related to metabolic syndrome, and anti-inflammatory activity of leaf extract from different Momordica species. Food Sci. Hum. Wellness.

[37] Thiruvengadam M., Chung I.M. (2015). Phenolic compound production and biological activities from in vitro regenerated plants of gherkin (*Cucumis anguria* L.). Electron. J. Biotechnol..

[38] Singh J., Singh V., Shukla S., Rai A.K. (2016). Phenolic Content and Antioxidant Capacity of Selected Cucurbit Fruits Extracted with Different Solvents. J. Nutr. Food Sci..

[39] Abbas H.M.K., Huang H.X., Huang W.J., Xue S.D., Yan S.J., Wu T.Q., Li J.X., Zhong Y.J. (2020). Evaluation of Metabolites and Antioxidant Activity in Pumpkin Species. Nat. Prod. Commun..

[40] Yadav M., Jain S., Tomar R., Prasad G.B.K.S., Yadav H. (2010). Medicinal and biological potential of pumpkin. An updated review. Nutr. Res. Rev..

[41] Caili F., Huan S., Quanhojg L. (2006). A Review on Pharmacological Activities and Utilization Technologies of Pumpkin. Plant Foods Hum. Nutr..

[42] Boiteux L.S., Nascimento W.M., Fonseca M.E.d.N., Lana M.M., Reis A., Mendonça J.L., Lopes J.F., Reifschneider F.J.B. (2007). “Brasileirinha”: An ornamental bicolor squash (*Cucurbita moschata*) cultivar for immature fruit consumption. Hortic. Bras..

[43] Rodriguez-Amaya D.B., Kimura M., Godoy H.T., Amaya-Farfan J. (2008). Updated Brazilian database on food carotenoids. Factors affecting carotenoid composition. J. Food Compos. Anal..

[44] Shi J., Yi C., Ye X., Xue S., Jiang Y., Ma Y., Liu D. (2010). Effects of supercritical CO_2_ fluid parameters on chemical composition and yield of carotenoids extracted from pumpkin. LWT Food Sci. Technol..

[45] Durante M., Lenucci M.S., D’Amico L., Piro G., Mita G. (2014). Effect of drying and co-matrix addition on the yield and quality of supercritical CO_2_ extracted pumpkin (*Cucurbita moschata* Duch.) oil. Food Chem..

[46] Murkovic M., Mülleder U., Neunteufl H. (2002). Carotenoid content in different varieties of pumpkins. J. Food Compos. Anal..

[47] Shi X., Wu H., Shi J., Xue S.J., Wang D., Wang W., Cheng A., Gong Z., Chen X., Wang C. (2013). Effect of modifier on the composition and antioxidant activity of carotenoid extracts from pumpkin (*Cucurbita maxima*) by supercritical CO_2_. LWT Food Sci. Technol..

[48] Wang S.-M., Yu D.-J., Song K.B. (2011). Physicochemical property of pumpkin slices dehydrated with red algae extract. J. Korean Soc. Appl. Biol. Chem..

[49] Nawirska A., Figiel A., Kucharska A.Z., Sokól-Letowska A., Biesiada A. (2009). Drying kinetics and quality parameters of pumpkin slices dehydrated using different methods. J. Food Eng..

[50] Fruhwirth G.O., Hermetter A. (2007). Seeds and oil of the Styrian oil pumpkin: Components and biological activities. Eur. J. Lipid Sci. Technol..

[51] Seo J.S., Burri B.J., Quan Z., Neidlinger T.R. (2005). Extraction and chromatography of carotenoids from pumpkin. J. Chromatogr. A.

[52] Akwaowo E.U., Ndon B.A., Etuk E.U. (2000). Minerals and antinutrients in fluted pumpkin (*Telfairia occidentalis* Hook f.). Food Chem..

[53] Montesano D., Rocchetti G., Putnik P., Lucini L. (2018). Bioactive profile of pumpkin: An overview on terpenoids and their health-promoting properties. Curr. Opin. Food Sci..

[54] Durante M., Lenucci M.S., Mita G. (2014). Supercritical carbon dioxide extraction of carotenoids from pumpkin (*Cucurbita* spp.): A review. Int. J. Mol. Sci..

[55] Perez Gutierrez R.M., Ramirez A.M., Mota Flores J.M., Garcia Campoy A.H. (2020). Antioxidant activity of a new multiflorane-type triterpene from *Cucurbita argyrosperma* seeds and their protective role in hydrogen peroxide induced oxidative stress. Funct. Foods Health Dis..

[56] Xia T., Wang Q. (2007). Hypoglycaemic role of *Cucurbita ficifolia* (Cucurbitaceae) fruit extract in streptozotocin-induced diabetic rats. J. Sci. Food Agric..

[57] Salazar R., Pozos M.E., Cordero P., Perez J., Salinas M.C., Waksman N. (2008). Determination of the Antioxidant Activity of Plants from Northeast Mexico. Pharm. Biol..

[58] Levi A., Jarret R., Kousik S., Patrick Wechter W., Nimmakayala P., Reddy U.K., Grumet R., Katzir N., Garcia-Mas J. (2017). Genetic Resources of Watermelon. Genetics, Genomics and Breeding of Cucurbits.

[59] Achigan-Dako E.G., Fagbemissi R., Avohou E.T., Vodouhe R.S., Coulibaly O., Ahanchede A. (2008). Importance and practices of Egusi crops (*Citrullus lanatus* (Thunb.) Matsum. & Nakai, *Cucumeropsis mannii* Naudin and *Lagenaria siceraria* (Molina) Standl. cv. ‘Aklamkpa’) in sociolinguistic areas in Benin. Biotechnol. Agron. Société Environ. Biotechnol. Agron. Soc. Environ..

[60] Bush A. (1978). Citron melon for cash and condiment. Econ. Bot..

[61] Hussain A.I., Rathore H.A., Sattar M.Z.A., Chatha S.A.S., Sarker S.D., Gilani A.H. (2014). *Citrullus colocynthis* (L.) Schrad (bitter apple fruit): A review of its phytochemistry, pharmacology, traditional uses and nutritional potential. J. Ethnopharmacol..

[62] Abdelwahab S.I., Hassan L.E.A., Sirat H.M., Yagi S.M.A., Koko W.S., Mohan S., Taha M.M.E., Ahmad S., Chuen C.S., Narrima P. (2011). Anti-inflammatory activities of cucurbitacin e isolated from *Citrullus lanatus* var. *citroides*: Role of reactive nitrogen species and cyclooxygenase enzyme inhibition. Fitoterapia.

[63] Adetutu A., Olorunnisola O.S., Owoade O.A. (2015). Nutritive Values and Antioxidant Activity of *Citrullus lanatus* Fruit Extract. Food Nutr. Sci..

[64] Jin B., Lee J., Kweon S., Cho Y., Choi Y., Lee S.J., Park Y. (2019). Analysis of flesh color-related carotenoids and development of a *CRTISO* gene-based DNA marker for prolycopene accumulation in watermelon. Hortic. Environ. Biotechnol..

[65] Liu C., Zhang H., Dai Z., Liu X., Liu Y., Deng X., Chen F., Xu J. (2012). Volatile chemical and carotenoid profiles in watermelons *Citrullus vulgaris* (Thunb.) Schrad (Cucurbitaceae) with different flesh colors. Food Sci. Biotechnol..

[66] Perkins-Veazie P., Collins J.K., Davis A.R., Roberts W. (2006). Carotenoid content of 50 watermelon cultivars. J. Agric. Food Chem..

[67] Tlili I., Hdider C., Lenucci M.S., Riadh I., Jebari H., Dalessandro G. (2011). Bioactive compounds and antioxidant activities of different watermelon (*Citrullus lanatus* (Thunb.) Mansfeld) cultivars as affected by fruit sampling area. J. Food Compos. Anal..

[68] Perkins-Veazie P., Maness N., Roduner R. (2002). Composition of orange, yellow, and red-fleshed watermelons. Cucurbitaceae.

[69] Rimando A.M., Perkins-Veazie P.M. (2005). Determination of citrulline in watermelon rind. J. Chromatogr. A.

[70] Yuan P., He N., Umer M.J., Zhao S., Diao W., Zhu H., Dou J., Kaseb M.O., Kuang H., Lu X. (2021). Comparative Metabolomic Profiling of *Citrullus* spp. Fruits Provides Evidence for Metabolomic Divergence during Domestication. Metabolites.

[71] Tarazona-Díaz M.P., Viegas J., Moldao-Martins M., Aguayo E. (2011). Bioactive compounds from flesh and by-product of fresh-cut watermelon cultivars. J. Sci. Food Agric..

[72] Davis A.R., Webber C.L., Fish W.W., Wehner T.C., King S., Perkins-Veazie P. (2011). L-citrulline levels in watermelon cultigens tested in two environments. HortScience.

[73] Emmanuel A.M., Phatlane W.M., Phetole M. (2018). Comparative analysis of the chemical compositions of indigenous watermelon (*Citrullus lanatus*) seeds from two districts in Limpopo Province, South Africa. Afr. J. Biotechnol..

[74] Sathya J., Shoba F.G. (2014). A study on the phytochemistry and antioxidant effect of methanolic extract of *Citrullus lanatus* seed. Pelagia Res. Libr. Asian J. Plant Sci. Res..

[75] Siddhuraju P., Mohan P.S., Becker K. (2002). Studies on the antioxidant activity of Indian Laburnum (*Cassia fistula* L.): A preliminary assessment of crude extracts from stem bark, leaves, flowers and fruit pulp. Food Chem..

[76] Hussain A.I., Rathore H.A., Sattar M.Z.A., Chatha S.A.S., Ahmad F., Ahmad A., Johns E.J. (2013). Phenolic profile and antioxidant activity of various extracts from *Citrullus colocynthis* (L.) from the Pakistani flora. Ind. Crop. Prod..

[77] Salandanan K., Bunning M., Stonaker F., Külen O., Kendall P., Stushnoff C. (2009). Comparative analysis of antioxidant properties and fruit quality attributes of organically and conventionally grown melons (*Cucumis melo* L.). HortScience.

[78] Ganji S.M., Singh H., Friedman M. (2019). Phenolic Content and Antioxidant Activity of Extracts of 12 Melon (*Cucumis melo*) Peel Powders Prepared from Commercial Melons. J. Food Sci..

[79] Vouldoukis I., Lacan D., Kamate C., Coste P., Calenda A., Mazier D., Conti M., Dugas B. (2004). Antioxidant and anti-inflammatory properties of a *Cucumis melo* LC. extract rich in superoxide dismutase activity. J. Ethnopharmacol..

[80] Ismail H.I., Chan K.W., Mariod A.A., Ismail M. (2010). Phenolic content and antioxidant activity of cantaloupe (*Cucumis melo*) methanolic extracts. Food Chem..

[81] Pyo Y.H., Lee T.C., Logendra L., Rosen R.T. (2004). Antioxidant activity and phenolic compounds of Swiss chard (*Beta vulgaris* subspecies *cycla*) extracts. Food Chem..

[82] Wong P.Y.Y., Kitts D.D. (2006). Studies on the dual antioxidant and antibacterial properties of parsley (*Petroselinum crispum*) and cilantro (*Coriandrum sativum*) extracts. Food Chem..

[83] Vella F.M., Cautela D., Laratta B. (2019). Characterization of Polyphenolic Compounds in Cantaloupe Melon By-Products. Foods.

[84] Melo E.D.A., Lima V.L.A.G., Maciel M.I.S., Caetano A.D.S., Leal F.L.L. (2006). Polyphenol, ascorbic acid and total carotenoid contents in common fruits and vegetables. Braz. J. Food Technol..

[85] Yunusa A.K., Dandago M.A., Ibrahim S.M., Abdullahi N., Rilwan A., Barde A. (2019). Total Phenolic Content and Antioxidant Capacity of Different Parts of Cucumber (*Cucumis sativus* L.). Acta Univ. Cibiniensis Ser. E Food Technol..

[86] Abifarin T.O., Afolayan A.J., Otunola G.A. (2019). Phytochemical and Antioxidant Activities of *Cucumis africanus* L.f.: A Wild Vegetable of South Africa. J. Evid. Based Integr. Med..

[87] Lee S.H., Jeong Y.S., Song J., Hwang K.-A., Noh G.M., Hwang I.G. (2017). Phenolic acid, carotenoid composition, and antioxidant activity of bitter melon (*Momordica charantia* L.) at different maturation stages. Int. J. Food Prop..

[88] Bruno A., Durante M., Marrese P.P., Migoni D., Laus M.N., Pace E., Pastore D., Mita G., Piro G., Lenucci M.S. (2018). Shades of red: Comparative study on supercritical CO_2_ extraction of lycopene-rich oleoresins from gac, tomato and watermelon fruits and effect of the α-cyclodextrin clathrated extracts on cultured lung adenocarcinoma cells’ viability. J. Food Compost. Anal..

[89] Ishida B.K., Turner C., Chapman M.H., McKeon T.A. (2004). Fatty Acid and Carotenoid Composition of Gac (*Momordica cochinchinensis* Spreng) Fruit. J. Agric. Food Chem..

[90] Vuong L.T., Franke A.A., Custer L.J., Murphy S.P. (2006). *Momordica cochinchinensis* Spreng. (gac) fruit carotenoids reevaluated. J. Food Compos. Anal..

[91] Kubola J., Siriamornpun S. (2008). Phenolic contents and antioxidant activities of bitter gourd (*Momordica charantia* L.) leaf, stem and fruit fraction extracts in vitro. Food Chem..

[92] Aoki H., Thi Minh Kieu N., Kuze N., Tomisaka K., Van Chuyen N. (2002). Bioscience, Biotechnology, and Biochemistry Carotenoid Pigments in GAC Fruit (*Momordica cochinchinensis* Spreng). Biotechnol. Biochem..

[93] Mikulic-Petkovsek M., Schmitzer V., Slatnar A., Stampar F., Veberic R. (2012). Composition of Sugars, Organic Acids, and Total Phenolics in 25 Wild or Cultivated Berry Species. J. Food Sci..

[94] Choo W.S., Yap J.Y., Chan S.Y. (2014). Antioxidant Properties of Two Varieties of Bitter Gourd (*Momordica charantia*) and the Effect of Blanching and Boiling on Them. Pertanika J. Trop. Agric. Sci..

[95] Iqbal M.P., Kazim S.F., Mehboobali N. (2006). Ascorbic acid contents of Pakistani fruits and vegetables. Pak. J. Pharm. Sci..

[96] Myojin C., Enami N., Nagata A., Yamaguchi T., Takamura H., Matoba T. (2008). Changes in the radical-scavenging activity of bitter gourd (*Momordica charantia* L.) during freezing and frozen storage with or without blanching. J. Food Sci..

[97] Somsub W., Kongkachuichai R., Sungpuag P., Charoensiri R. (2008). Effects of three conventional cooking methods on vitamin C, tannin, myo-inositol phosphates contents in selected Thai vegetables. J. Food Compos. Anal..

[98] Patel S.B., Attar U.A., Ghane S.G. (2018). Antioxidant potential of wild *Lagenaria siceraria* (Molina) Standl. Thai J. Pharm. Sci..

[99] Sharma N.K., Yadav P., Kumar Singh H., Shrivastava A.K. (2013). In vitro antioxidant activity of *Lagenaria siceraria* leaves. Malays. J Pharm. Sci..

[100] Wang H.X., Ng T.B. (2000). Lagenin, a novel ribosome-inactivating protein with ribonucleolytic activity from bottle gourd (*Lagenaria siceraria*) seeds. Life Sci..

[101] Khare C.P. (2004). Indian Herbal Remedies: Rational Western Therapy, Ayurvedic and Other Traditional Usage, Botany.

[102] Ahmed D., Fatima M., Saeed S. (2014). Phenolic and flavonoid contents and anti-oxidative potential of epicarp and mesocarp of *Lagenaria siceraria* fruit: A comparative study. Asian Pac. J. Trop. Med..

[103] Agrawal S., Pg G.K.R.G., Prasad G. (2015). Antioxidant Activity, Total Phenolic Compound and Flavonoid Content of Vaccum Dreid Extract of *L. siceraria* Charu Katare. Glob. J. Multidiscip. Stud..

[104] Mohan R., Birari R., Karmase A., Jagtap S., Bhutani K.K. (2012). Antioxidant activity of a new phenolic glycoside from *Lagenaria siceraria* Stand. fruits. Food Chem..

[105] Sabo H., Sadou H., Alma M.M., Sidikou R.S., Saadou M., Amoukou I.A. (2013). Antioxidant Activity and Phenolics Content of the Seeds of Eighteen Varieties of Edible Cucurbitaceae of Niger. J. Food Resour. Sci..

[106] Du Q., Xu Y., Li L., Zhao Y., Jerz G., Winterhalter P. (2006). Antioxidant Constituents in the Fruits of *Luffa cylindrica* (L.) Roem. J. Agric. Food Chem..

[107] Swetha M.P., Muthukumar S.P. (2016). Characterization of nutrients, amino acids, polyphenols and antioxidant activity of Ridge gourd (*Luffa acutangula*) peel. J. Food Sci. Technol..

[108] Suryanti V., Marliyana S.D., Wulandari T. (2015). Antioxidant activity, total phenolics and flavonoids contents of *Luffa acutangula* (L.) Roxb fruit. J. Chem. Pharm. Res..

[109] Siciliano T., De Tommasi N., Morelli I., Braca A. (2004). Study of Flavonoids of *Sechium edule* (Jacq) Swartz (Cucurbitaceae) Different Edible Organs by Liquid Chromatography Photodiode Array Mass Spectrometry. J. Agric. Food Chem..

[110] Lachman J., Šulc M., Faitová K., Pivec V. (2009). Major factors influencing antioxidant contents and antioxidant activity in grapes and wines. Int. J. Wine Res..

[111] Kalt W., Prange R.K., Lidster P.D. (1993). Postharvest color development of strawberries: Influence of maturity, temperature and light. Can. J. Plant Sci..

[112] Kalt W. (2005). Effects of Production and Processing Factors on Major Fruit and Vegetable Antioxidants. J. Food Sci..

[113] Jakopic J., Stampar F., Veberic R. (2009). The influence of exposure to light on the phenolic content of “Fuji” apple. Sci. Hortic..

[114] Tsormpatsidis E., Henbest R.G.C., Battey N.H., Hadley P. (2010). The influence of ultraviolet radiation on growth, photosynthesis and phenolic levels of green and red lettuce: Potential for exploiting effects of ultraviolet radiation in a production system. Ann. Appl. Biol..

[115] Quintero-Arias D.G., Acuña-Caita J.F., Asensio C., Valenzuela J.L. (2021). Ultraviolet Transparency of Plastic Films Determines the Quality of Lettuce (*Lactuca sativa*, L.) Grown in a Greenhouse. Agronomy.

[116] Martínez-Zamora L., Castillejo N., Gómez P.A., Artés-Hernández F. (2021). Amelioration Effect of LED Lighting in the Bioactive Compounds Synthesis during Carrot Sprouting. Agronomy.

[117] Palma C.F.F., Castro-Alves V., Morales L.F., Rosenqvist E., Ottosen C.-O., Strid A. (2021). Spectral Composition of Light Affects Sensitivity to UV-B and Photoinhibition in Cucumber. Front. Plant Sci..

[118] Mastropasqua L., Dipierro N., Paciolla C. (2020). Effects of Darkness and Light Spectra on Nutrients and Pigments in Radish, Soybean, Mung Bean and Pumpkin Sprouts. Antioxidants.

[119] Hartman J., Wehner T., Ma G., Perkins-Veazie P. (2019). Citrulline and Arginine Content of Taxa of Cucurbitaceae. Horticulturae.

[120] Bisognin D.A. (2002). Origin and evolution of cultivated cucurbits. Ciência Rural.

[121] Ara N., Nakkanong K., Lv W., Yang J., Hu Z., Zhang M. (2013). Antioxidant enzymatic activities and gene expression associated with heat tolerance in the stems and roots of two cucurbit species (“*Cucurbita maxima*” and “*Cucurbita moschata*”) and their interspecific inbred line “Maxchata”. Int. J. Mol. Sci..

[122] Seki M., Kamei A., Yamaguchi-Shinozaki K., Shinozaki K. (2003). Molecular responses to drought, salinity and frost: Common and different paths for plant protection. Curr. Opin. Biotechnol..

[123] Mukherjee P.K., Nema N.K., Maity N., Sarkar B.K. (2013). Phytochemical and therapeutic potential of cucumber. Fitoterapia.

[124] Mashilo J., Odindo A.O., Shimelis H.A., Musenge P., Tesfay S.Z., Magwaza L.S. (2018). Photosynthetic response of bottle gourd [*Lagenaria siceraria* (Molina) Standl.] to drought stress: Relationship between cucurbitacins accumulation and drought tolerance. Sci. Hortic..

[125] Fan H.F., Ding L., Xu Y.L., Du C.X. (2017). Antioxidant System and Photosynthetic Characteristics Responses to Short-term PEG-induced Drought Stress in Cucumber Seedling Leaves. Russ. J. Plant Physiol..

[126] Hamurcu M., Khan M.K., Pandey A., Ozdemir C., Avsaroglu Z.Z., Elbasan F., Omay A.H., Gezgin S. (2020). Nitric oxide regulates watermelon (*Citrullus lanatus*) responses to drought stress. 3 Biotech.

[127] Hasanuzzaman M., Oku H., Nahar K., Bhuyan M.B., Al Mahmud J., Baluska F., Fujita M. (2018). Nitric oxide-induced salt stress tolerance in plants: ROS metabolism, signaling, and molecular interactions. Plant Biotechnol. Rep..

[128] Mittova V., Tal M., Volokita M., Guy M. (2003). Up-regulation of the leaf mitochondrial and peroxisomal antioxidative systems in response to salt-induced oxidative stress in the wild salt-tolerant tomato species *Lycopersicon pennellii*. Plant Cell Environ..

[129] Furtana G.B., Tipirdamaz R. (2010). Physiological and antioxidant response of three cultivars of cucumber (*Cucumis sativus* L.) to salinity. Turk. J. Biol..

[130] Erdinc C. (2018). Changes in ion (K, Ca and Na) regulation, antioxidant enzyme activity and photosynthetic pigment content in melon genotypes subjected to salt stress–a mixture modeling analysis. Acta Sci. Pol. Hortorum Cultus.

[131] KeLing H., Ling Z., JiTao W., Yang Y. (2013). Influence of selenium on growth, lipid peroxidation and antioxidative enzyme activity in melon (*Cucumis melo* L.) seedlings under salt stress. Acta Soc. Bot. Pol..

[132] Kusvuran S., Ellialtioglu S., Yasar F., Abak K., Rastilantie M. (2007). Effects of salt stress on ion accumulation and activity of some antioxidant enzymes in melon (Cucumis melo L.). J. Food Agric. Environ..

[133] Sarabi B., Bolandnazar S., Ghaderi N., Ghashghaie J. (2017). Genotypic differences in physiological and biochemical responses to salinity stress in melon (*Cucumis melo* L.) plants: Prospects for selection of salt tolerant landraces. Plant Physiol. Biochem..

[134] Toscano S., Trivellini A., Cocetta G., Bulgari R., Francini A., Romano D., Ferrante A. (2019). Effect of Preharvest Abiotic Stresses on the Accumulation of Bioactive Compounds in Horticultural Produce. Front. Plant Sci..

[135] Wang S.Y., Zheng W. (2005). Preharvest application of methyl jasmonate increases fruit quality and antioxidant capacity in raspberries. Int. J. Food Sci. Technol..

[136] Giménez M.J., Serrano M., Valverde J.M., Martínez-Romero D., Castillo S., Valero D., Guillén F. (2017). Preharvest salicylic acid and acetylsalicylic acid treatments preserve quality and enhance antioxidant systems during postharvest storage of sweet cherry cultivars. J. Sci. Food Agric..

[137] Khalil H.A. (2020). Improved Yield, Fruit Quality, and Shelf Life in “Flame Seedless” Grapevine with Pre-Harvest Foliar Applications of Forchlorfenuron, Gibberellic Acid, and Abscisic Acid. J. Hortic. Res..

[138] Lobos T.E., Retamales J.B., Hanson E.J. (2021). Early preharvest calcium sprays improve postharvest fruit quality in ‘Liberty’ highbush blueberries. Sci. Hortic..

[139] Ge Y., Li X., Li C., Tang Q., Duan B., Cheng Y., Hou J., Li J. (2019). Effect of sodium nitroprusside on antioxidative enzymes and the phenylpropanoid pathway in blueberry fruit. Food Chem..

[140] Serrano M., Martínez-Esplá A., Zapata P., Castillo S., Martínez-Romero D., Guillén F., Valverde J.M., Valero D., Barman K., Sharma S., Siddiqui M.W. (2018). Effects of methyl jasmonate treatment on fruit quality properties. Emerging Postharvest Treatment of Fruits and Vegetables.

[141] Yuan M., Huang Y., Ge W., Jia Z., Song S., Zhang L., Huang Y. (2019). Involvement of jasmonic acid, ethylene and salicylic acid signaling pathways behind the systemic resistance induced by Trichoderma longibrachiatum H9 in cucumber. BMC Genom..

[142] Chan Z., Wang Q., Xu X., Meng X., Qin G., Li B., Tian S. (2008). Functions of defense-related proteins and dehydrogenases in resistance response induced by salicylic acid in sweet cherry fruits at different maturity stages. Proteomics.

[143] Martínez-Esplá A., Serrano M., Valero D., Martínez-Romero D., Castillo S., Zapata P. (2017). Enhancement of Antioxidant Systems and Storability of Two Plum Cultivars by Preharvest Treatments with Salicylates. Int. J. Mol. Sci..

[144] Asghari M., Aghdam M.S. (2010). Impact of salicylic acid on post-harvest physiology of horticultural crops. Trends Food Sci. Technol..

[145] Xue H., Bi Y., Sun Y., Hussain R., Wang H., Zhang S., Zhang R., Long H., Nan M., Cheng X. (2019). Acetylsalicylic acid treatment reduce Fusarium rot development and neosolaniol accumulation in muskmelon fruit. Food Chem..

[146] Wang B., Bai X., Zhang J., Wang Y., Jiang H., Bi Y. (2018). The induction of pre-harvest acetylsalicylic acid treatment on postharvest resistance of Hami melon fruit. J. Fruit Sci..

[147] Rehman H., Farooq M., Basra S.M.A., Afzal I. (2011). Hormonal priming with salicylic acid improves the emergence and early seedling growth in cucumber. J. Agric. Soc. Sci..

[148] Preciado-Rangel P., Reyes-Pérez J., Ramírez-Rodríguez S., Salas-Pérez L., Fortis-Hernández M., Murillo-Amador B., Troyo-Diéguez E. (2019). Foliar Aspersion of Salicylic Acid Improves Phenolic and Flavonoid Compounds, and Also the Fruit Yield in Cucumber (*Cucumis sativus* L.). Plants.

[149] Khan A.S., Ali S., Sidiqqui M.W. (2018). Preharvest sprays affecting shelf life and storage potential of fruits. Preharvest Modulation of Postharvest Fruit and Vegetable Quality.

[150] Massolo J.F., Lemoine M.L., Chaves A.R., Concellón A., Vicente A.R. (2014). Benzyl-aminopurine (BAP) treatments delay cell wall degradation and softening, improving quality maintenance of refrigerated summer squash. Postharvest Biol. Technol..

[151] Li Q., Yu B., Gao Y., Dai A.H., Bai J.G. (2011). Cinnamic acid pretreatment mitigates chilling stress of cucumber leaves through altering antioxidant enzyme activity. J. Plant Physiol..

[152] Lee J.M., Kubota C., Tsao S.J., Bie Z., Echevarria P.H., Morra L., Oda M. (2010). Current status of vegetable grafting. Diffusion, grafting techniques, automation. Sci. Hortic..

[153] Davis A.R., Perkins-Veazie P., Sakata Y., López-Galarza S., Maroto J.V., Lee S.-G., Huh Y.-C., Sun Z., Miguel A., King S.R. (2008). Cucurbit Grafting. CRC Crit. Rev. Plant Sci..

[154] King S.R., Davis A.R., Zhang X., Crosby K. (2010). Genetics, breeding and selection of rootstocks for solanaceae and cucurbitaceae. Sci. Hortic..

[155] Modarelli G.C., Rouphael Y., De Pascale S., Öztekin G.B., Tüzel Y., Orsini F., Gianquinto G. (2020). Appraisal of Salt Tolerance under Greenhouse Conditions of a Cucurbitaceae Genetic Repository of Potential Rootstocks and Scions. Agronomy.

[156] Kumar P., Rouphael Y., Cardarelli M., Colla G. (2017). Vegetable Grafting as a Tool to Improve Drought Resistance and Water Use Efficiency. Front. Plant Sci..

[157] Liu S., Li H., Lv X., Ahammed G.J., Xia X., Zhou J., Shi K., Asami T., Yu J., Zhou Y. (2016). Grafting cucumber onto luffa improves drought tolerance by increasing ABA biosynthesis and sensitivity. Sci. Rep..

[158] Alan O., Sen F., Duzyaman E. (2018). The effectiveness of growth cycle on improving fruit quality for grafted watermelon combinations. Food Sci. Technol..

[159] Fallik E., Ziv C. (2020). How rootstock/scion combinations affect watermelon fruit quality after harvest?. J. Sci. Food Agric..

[160] Fallik E., Alkalai-Tuvia S., Chalupowicz D., Popovsky-Sarid S., Zaaroor-Presman M. (2019). Relationships between Rootstock-Scion Combinations and Growing Regions on Watermelon Fruit Quality. Agronomy.

[161] Elsheery N.I., Helaly M.N., Omar S.A., John S.V.S., Zabochnicka-Swiątek M., Kalaji H.M., Rastogi A. (2020). Physiological and molecular mechanisms of salinity tolerance in grafted cucumber. S. Afr. J. Bot..

[162] Tao M.-Q., Jahan M.S., Hou K., Shu S., Wang Y., Sun J., Guo S.-R. (2020). Bitter Melon (*Momordica charantia* L.) Rootstock Improves the Heat Tolerance of Cucumber by Regulating Photosynthetic and Antioxidant Defense Pathways. Plants.

[163] Olguín M.A.V., Fuente la M.C.D., Mendoza A.B., Maldonado A.J., Rangel A.S., Cusimamani E.F. (2020). Commercial and nutraceutical quality of grafted melon cultivated under hydric stress. Hortic. Sci..

[164] Expósito A., Pujolà M., Achaerandio I., Giné A., Escudero N., Fullana A.M., Cunquero M., Loza-Alvarez P., Sorribas F.J. (2020). Tomato and Melon Meloidogyne Resistant Rootstocks Improve Crop Yield but Melon Fruit Quality Is Influenced by the Cropping Season. Front. Plant Sci..

[165] Oloyede F.M., Agbaje G.O., Obuotor E.M., Obisesan I.O. (2012). Nutritional and antioxidant profiles of pumpkin (Cucurbita pepo Linn.) immature and mature fruits as influenced by NPK fertilizer. Food Chem..

[166] Valenzuela J.L., Sanchez A., Romero L. (1994). Influence of nitrogen, phosphorus, and potassium fertilization on foliar pigments in muskmelon plants. Comm. Soil Sci. Plant Anal..

[167] Valenzuela J.L., Sanchez A., del Río A., Romero L. (1992). Relationships between nitrogen supply and different phosphorus and calcium fractions in leaves of tomato and cucumber plants. Israel J. Plant Sci..

[168] Kopczyńska K., Kazimierczak R., Średnicka-Tober D., Barański M., Wyszyński Z., Kucińska K., Perzanowska A., Szacki P., Rembiałkowska E., Hallmann E. (2020). The Profile of Selected Antioxidants in Two Courgette Varieties from Organic and Conventional Production. Antioxidants.

[169] Panuccio M.R., Papalia T., Attinà E., Giuffrè A., Muscolo A. (2019). Use of digestate as an alternative to mineral fertilizer: Effects on growth and crop quality. Arch. Agron. Soil Sci..

[170] Kevers C., Falkowski M., Tabart J., Defraigne J.O., Dommes J., Pincemail J. (2007). Evolution of antioxidant capacity during storage of selected fruits and vegetables. J. Agric. Food Chem..

[171] Tomás-Barberan F.A., Ferreres F., Gil M.I. (2000). Antioxidant phenolic metabolites from fruit and vegetables and changes during postharvest storage and processing. Stud. Nat. Prod. Chem..

[172] Lee S.K., Kader A.A. (2000). Preharvest and postharvest factors influencing vitamin C content of horticultural crops. Postharvest Biol. Technol..

[173] Megias Z., González-Rodríguez L.J., Aguado E., García A., Manzano S., Rebolloso M.M., Valenzuela J.L., Jamilena M. (2018). Effect of cold storage time on chilling injury in two zucchini cultivars. Acta Hortic..

[174] Hariyadi P., Parkin K.L. (1991). Chilling-induced oxidative stress in cucumber fruits. Postharvest Biol. Technol..

[175] Vanlerberghe G. (2013). Alternative Oxidase: A Mitochondrial Respiratory Pathway to Maintain Metabolic and Signaling Homeostasis during Abiotic and Biotic Stress in Plants. Int. J. Mol. Sci..

[176] Qian C., Mi H., Zhao Y., He Z., Mao L. (2013). Effect of Maturity Stage on the Gene Expression of Antioxidative Enzymes in Cucumber (*Cucumis sativus* L.) Fruits under Chilling Stress. J. Integr. Agric..

[177] Megías Z., Manzano S., Martínez C., García A., Aguado E., Garrido D., del Mar Rebolloso M., Valenzuela J.L., Jamilena M. (2017). Postharvest cold tolerance in summer squash and its association with reduced cold-induced ethylene production. Euphytica.

[178] García A., Aguado E., Cebrián G., Iglesias J., Romero J., Martínez C., Garrido D., del Rebolloso M., Valenzuela J.L., Jamilena M. (2020). Effect of Ethylene-Insensitive Mutation etr2b on Postharvest Chilling Injury in Zucchini Fruit. Agriculture.

[179] Wang C.Y. (1995). Effect of temperature preconditioning on catalase, peroxidase, and superoxide dismutase in chilled zucchini squash. Postharvest Biol. Technol..

[180] Carvajal F., Palma F., Jamilena M., Garrido D. (2015). Preconditioning treatment induces chilling tolerance in zucchini fruit improving different physiological mechanisms against cold injury. Ann. Appl. Biol..

[181] Megías Z., Barrera A., Manzano S., Martínez C., Garrido D., Valenzuela J.L., Jamilena M. (2015). Physical and chemical treatments enhancing postharvest fruit quality in zucchini. Acta Hortic..

[182] Ghaouth A., Arul J., Ponnampalam R., Boulet M. (1991). Use of chitosan coating to reduce water loss and maintain quality of cucumber and bell pepper fruits. J. Food Process. Preserv..

[183] Dhall R.K., Sharma S.R., Mahajan B.V.C. (2012). Effect of shrink wrap packaging for maintaining quality of cucumber during storage. J. Food Sci. Technol..

[184] Megías Z., Martínez C., Manzano S., García A., Del Mar Rebolloso-Fuentes M., Garrido D., Valenzuela J.L., Jamilena M. (2015). Individual shrink wrapping of zucchini fruit improves postharvest chilling tolerance associated with a reduction in ethylene production and oxidative stress metabolites. PLoS ONE.

[185] Artés-Hernández F., Robles P.A., Gómez P.A., Tomás-Callejas A., Artés F., Martínez-Hernández G.B. (2021). Quality Changes of Fresh-Cut Watermelon During Storage as Affected by Cut Intensity and UV-C Pre-treatment. Food Bioprocess Technol..

[186] Liu Y., Yang X., Zhu S., Wang Y. (2016). Postharvest application of MeJA and NO reduced chilling injury in cucumber (*Cucumis sativus*) through inhibition of H2O2 accumulation. Postharvest Biol. Technol..

[187] Xu T., Chen Y., Kang H. (2019). Melatonin Is a Potential Target for Improving Post-Harvest Preservation of Fruits and Vegetables. Front. Plant Sci..

[188] Xin D., Si J., Kou L. (2017). Postharvest exogenous melatonin enhances quality and delays the senescence of cucumber. Acta Hortic. Sin..

[189] Lin X., Wang L., Hou Y., Zheng Y., Jin P.A. (2020). Combination of Melatonin and Ethanol Treatment Improves Postharvest Quality in Bitter Melon Fruit. Foods.

[190] Aghdam M.S., Asghari M., Babalar M., Sarcheshmeh M.A.A., Siddiqui M.W. (2016). Impact of salicylic acid on postharvest physiology of fruits and vegetables. Eco-Friendly Technology for Postharvest Produce Quality.

[191] Han C., Zuo J.H., Qing W., Dong H.Z., Gao L.P. (2017). Salicylic acid alleviates postharvest chilling injury of sponge gourd (*Luffa cylindrica*). J. Integr. Agric..

[192] Zapata S., Carrera R., Manzano S., Megías Z., García A., Aguado E., Rebolloso M.M., Valenzuela J.L., Jamilena M. (2016). Efectos de los tratamientos de Metil Jasmonato y Ácido Salicílico en la reducción del daño por frío en calabacín. Actas Port. Hortic..

[193] Ru L., Jiang L., Wills R.B.H., Golding J.B., Huo Y., Yang H., Li Y. (2020). Chitosan oligosaccharides induced chilling resistance in cucumber fruit and associated stimulation of antioxidant and HSP gene expression. Sci. Hortic..

[194] Hasan M.U., Riaz R., Malik A.U., Khan A.S., Anwar R., Rehman R.N.U., Ali S. (2021). Potential of *Aloe vera* gel coating for storage life extension and quality conservation of fruits and vegetables: An overview. J. Food Biochem..

[195] Leopoldini M., Marino T., Russo N., Toscano M. (2004). Antioxidant Properties of Phenolic Compounds: H-Atom versus Electron Transfer Mechanism. J. Phys. Chem. A.

[196] Piazzon A., Vrhovsek U., Masuero D., Mattivi F., Mandoj F., Nardini M. (2012). Antioxidant Activity of Phenolic Acids and Their Metabolites: Synthesis and Antioxidant Properties of the Sulfate Derivatives of Ferulic and Caffeic Acids and of the Acyl Glucuronide of Ferulic Acid. J. Agric. Food Chem..

[197] Dasgupta A., Klein K. (2014). Antioxidants in Food, Vitamins and Supplements: Prevention and Treatment of Disease.

[198] Ferriol M., Picó B., Prohens J., Nuez F. (2008). Pumpkin and winter squash. Handbook of Plant Breeding, Vegetables I.

[199] Ratnayake R.S., Hurst P.L., Melton L.D. (2004). Influence of cultivar, storage and cooking on the mechanical properties of winter squash (*Cucurbita maxima*). J. Sci. Food Agric..

[200] Baggett J.R., Kean D. (2019). Sugar Loaf and ‘Honey Boat’ Winter Squashes. HortScience.

[201] Wessel-Beaver L. (2005). Cultivar and germplasm release—Release of ‘Soler’ tropical pumpkin. J. Agric. Univ. Puerto Rico.

[202] Holmes A.D., Spelman A.F., Jones C.P. (1945). Ascorbic acid, carotene, chlorophyll, riboflavin, and water content of summer squashes. J. Food Sci..

[203] Borenstein B., Bunnell R.H. (1966). Carotenoids: Properties, Occurrence, and Utilization in Foods. Adv. Food Res..

[204] Paris H.S., Linskens H.S., Jackson J.F. (1994). Genetic Analysis and Breeding of Pumpkins and Squash for High Carotene Content. Vegetable and Vegetable Products.

[205] Lewis E., Merrow S. (1962). Influence on the estimation of β-carotene by other carotenoids in butternut squashes at harvest and during storage. J. Agric. Food Chem..

[206] Azevedo-Meleiro C.H., Rodriguez-Amaya D.B. (2007). Qualitative and Quantitative Differences in Carotenoid Composition among *Cucurbita moschata*, *Cucurbita maxima*, and *Cucurbita pepo*. J. Agric. Food Chem..

[207] Kaźmińska K., Hallmann E., Rusaczonek A., Korzeniewska A., Sobczak M., Filipczak J., Kuczerski K.S., Steciuk J., Sitarek-Andrzejczyk M., Gajewski M. (2018). Genetic mapping of ovary colour and quantitative trait loci for carotenoid content in the fruit of *Cucurbita maxima* Duchesne. Mol. Breed..

[208] Zhong Y.J., Zhou Y.Y., Li J.X., Yu T., Wu T.Q., Luo J.N., Luo S.B., Huang H.X. (2017). A high-density linkage map and QTL mapping of fruit-related traits in pumpkin (*Cucurbita moschata* Duch.). Sci. Rep..

[209] Paris H.S., Brown R.N. (2005). The Genes of Pumpkin and Squash. HortScience.

[210] Paris H.S., Prohens J., Nuez F. (2008). Summer squash. Handbook of Plant Breeding, Vegetables I.

[211] Paris H.S. (1988). Complementary genes for orange fruit flesh color in *Cucurbita pepo*. HortScience.

[212] Schaffer A.A., Paris H.S., Ascarelli I.M. (1986). Carotenoid and starch content of near-isogenic *B + B+* and *BB* genotypes of *Cucurbita*. J. Am. Soc Hort. Sci..

[213] Levi A., Thomas C.E. (2001). Low genetic diversity indicates the need to broaden the genetic base of cultivated watermelon. HortScience.

[214] Navazio J.P., Simon P.W. (2001). Diallel analysis of high carotenoid content in cucumbers. J. Am. Soc. Hortic. Sci..

[215] Li B., Lu X., Dou J., Aslam A., Gao L., Zhao S., He N., Liu W. (2018). Construction of A High-Density Genetic Map and Mapping of Fruit Traits in Watermelon (*Citrullus lanatus* L.) Based on Whole-Genome Resequencing. Int. J. Mol. Sci..

[216] Guo S., Zhao S., Sun H., Wang X., Wu S., Lin T., Ren Y., Gao L., Deng Y., Zhang J. (2019). Resequencing of 414 cultivated and wild watermelon accessions identifies selection for fruit quality traits. Nat. Genet..

[217] Mayer A.M. (2006). Polyphenol oxidases in plants and fungi: Going places? A review. Phytochemistry.

[218] Liu S., Gao P., Zhu Q., Luan F., Davis A.R., Wang X. (2016). Development of cleaved amplified polymorphic sequence markers and a CAPS-based genetic linkage map in watermelon (*Citrullus lanatus* [Thunb.] Matsum. and Nakai) constructed using whole-genome re-sequencing data. Breed. Sci..

[219] Bang H., Kim S., Leskovar D., King S. (2007). Development of a codominant CAPS marker for allelic selection between canary yellow and red watermelon based on SNP in *lycopene β-cyclase* (*LCYB*) gene. Mol. Breed..

[220] Branham S., Vexler L., Meir A., Tzuri G., Frieman Z., Levi A., Wechter W.P., Tadmor Y., Gur A. (2017). Genetic mapping of a major codominant QTL associated with β-carotene accumulation in watermelon. Mol. Breed..

[221] Sun T., Yuan H., Cao H., Yazdani M., Tadmor Y., Li L. (2018). Carotenoid Metabolism in Plants: The Role of Plastids. Mol. Plant.

[222] Guo S., Sun H., Zhang H., Liu J., Ren Y., Gong G., Jiao C., Zheng Y., Yang W., Fei Z. (2015). Comparative Transcriptome Analysis of Cultivated and Wild Watermelon during Fruit Development. PLoS ONE.

[223] Hughes M.B. (1948). The inheritance of two characters of *Cucumis melo* and their interrelationship. Proc. Soc. Hortic. Sci..

[224] Ramaswamy B., Seshadri V.S., Sharma J.C. (1977). Inheritance of some fruit characters in muskmelon. Sci. Hortic..

[225] Monforte A.J., Oliver M., Gonzalo M.J., Alvarez J.M., Dolcet-Sanjuan R., Arús P. (2004). Identification of quantitative trait loci involved in fruit quality traits in melon (*Cucumis melo* L.). Theor. Appl. Genet..

[226] Périn C., Hagen L.S., De Conto V., Katzir N., Danin-Poleg Y., Portnoy V., Baudracco-Arnas S., Chadoeuf J., Dogimont C., Pitrat M. (2002). A reference map of *Cucumis melo* based on two recombinant inbred line populations. Theor. Appl. Genet..

[227] Cuevas H.E., Staub J.E., Simon P.W., Zalapa J.E. (2009). A consensus linkage map identifies genomic regions controlling fruit maturity and beta-carotene-associated flesh color in melon (*Cucumis melo* L.). Theor. Appl. Genet..

[228] Gonzalo M.J., Monforte A.J., Grumet R., Katzir N., Garcia-Mas J. (2017). Genetic Mapping of Complex Traits in Cucurbits. Genetics, Genomics and Breeding of Cucurbits.

[229] Chayut N., Yuan H., Ohali S., Meir A., Sa’ar U., Tzuri G., Zheng Y., Mazourek M., Gepstein S., Zhou X. (2017). Distinct mechanisms of the ORANGE protein in controlling carotenoid flux. Plant Physiol..

[230] Tzuri G., Zhou X., Chayut N., Yuan H., Portnoy V., Meir A., Sa’ar U., Baumkoler F., Mazourek M., Lewinsohn E. (2015). A ‘golden’ SNP in *CmOr* governs the fruit flesh color of melon (*Cucumis melo*). Plant J..

[231] Cuevas H.E., Staub J.E., Simon P.W., Zalapa J.E., McCreight J.D. (2008). Mapping of genetic loci that regulate quantity of beta-carotene in fruit of US Western Shipping melon (*Cucumis melo* L.). Theor. Appl. Genet..

[232] Harel-Beja R., Tzuri G., Portnoy V., Lotan-Pompan M., Lev S., Cohen S., Dai N., Yeselson L., Meir A., Libhaber S.E. (2010). A genetic map of melon highly enriched with fruit quality QTLs and EST markers, including sugar and carotenoid metabolism genes. Theor. Appl. Genet..

[233] Pereira L., Ruggieri V., Pérez S., Alexiou K.G., Fernández M., Jahrmann T., Pujol M., Garcia-Mas J. (2018). QTL mapping of melon fruit quality traits using a high-density GBS-based genetic map. BMC Plant Biol..

[234] Cuevas H.E., Song H., Staub J.E., Simon P.W. (2010). Inheritance of beta-carotene-associated flesh color in cucumber (*Cucumis sativus* L.) fruit. Euphytica.

[235] Simon P.W., Navazio J.P. (1997). Early orange mass 400, early orange mass 402, and late orange mass 404: High-carotene cucumber germplasm. HortScience.

[236] Bo K., Song H., Shen J., Qian C., Staub J.E., Simon P.W., Lou Q., Chen J. (2012). Inheritance and mapping of theoregene controllingthe quantity of b-carotene in cucumber (*Cucumis sativus* L.) endocarp. Mol. Breed..

[237] Lu H.W., Miao H., Tian G.L., Wehner T.C., Gu X.F., Zhang S.P. (2015). Molecular mapping and candidate gene analysis for yellow fruit flesh in cucumber. Paleontol. J..

[238] Boualem A., Fleurier S., Troadec C., Audigier P., Kumar A.P.K., Chatterjee M., Alsadon A.A., Sadder M.T., Wahb-Allah M.A., Al-Doss A.A. (2014). Development of a *Cucumis sativus* TILLinG Platform for Forward and Reverse Genetics. PLoS ONE.

[239] Karmakar P., Munshi A.D., Behera T.K., Kumar R., Sureja A.K., Kaur C., Singh B.K. (2013). Quantification and Inheritance of Antioxidant Properties and Mineral Content in Ridge Gourd (*Luffa acutangula*). Agric. Res..

[240] Xue C., Wang C., Fan L., Hao N., Zou D., Zhang Q., Chen H., Wu T. (2016). Ethylmethanesulfonate mutagenesis of cucumber for large-scale mutant screens. Pak. J. Bot..

[241] Chen C., Cui Q., Huang S.W., Wang S.H., Liu X.H., Lu X.Y., Chen H.M., Tian Y. (2018). An EMS mutant library for cucumber. J. Integr. Agric..

[242] Tadmor Y., Katzir N., Meir A., Yaniv-Yaakov A., Sa’ar U., Baumkoler F., Lavee T., Lewinsohn E., Schaffer A., Burger J. (2007). Induced mutagenesis to augment the natural genetic variability of melon (*Cucumis melo* L.). Israel J. Plant Sci..

[243] González M., Xu M., Esteras C., Roig C., Monforte A.J., Troadec C., Pujol M., Nuez F., Bendahmane A., Garcia-Mas J. (2011). Towards a tilling platform for functional genomics in Piel de Sapo melons. BMC Res. Notes.

[244] Velkov N., Tomlekova N., Sarsu F. (2016). Sensitivity of watermelon variety Bojura to mutant agents 60 Co and EMS. BioSci. Biotechnol..

[245] Vicente-Dólera N., Troadec C., Moya M., Del Río-Celestino M., Pomares-Viciana T., Bendahmane A., Picó B., Román B., Gómez P. (2014). First TILLING platform in *Cucurbita pepo*: A new mutant resource for gene function and crop improvement. PLoS ONE.

[246] García A., Aguado E., Parra G., Manzano S., Martínez C., Megías Z., Cebrián G., Romero J., Beltrán S., Garrido D. (2018). Phenomic and Genomic Characterization of a Mutant Platform in *Cucurbita pepo*. Front. Plant Sci..

[247] Chen K., Wang Y., Zhang R., Zhang H., Gao C. (2019). CRISPR/Cas Genome Editing and Precision Plant Breeding in Agriculture. Annu. Rev. Plant Biol..

[248] Galpaz N., Burger Y., Lavee T., Tzuri G., Sherman A., Melamed T., Eshed R., Meir A., Portnoy V., Bar E. (2013). Genetic and chemical characterization of an EMS induced mutation in *Cucumis melo CRTISO* gene. Arch. Biochem. Biophys..

[249] Tian S., Jiang L., Gao Q., Zhang J., Zong M., Zhang H., Ren Y., Guo S., Gong G., Liu F. (2017). Efficient CRISPR/Cas9-based gene knockout in watermelon. Plant Cell Rep..

[250] Zhang Y., Chu G., Hu Z., Gao Q., Cui B., Tian S., Wang B., Chen G. (2016). Genetically engineered anthocyanin pathway for high health-promoting pigment production in eggplant. Mol. Breed..

[251] Jang H.A., Utomo S.D., Kwon S.Y., Ha S.H., Ye X.-G., Choi P.S. (2016). Production of transgenic cucumber expressing *phytoene synthase-2A carotene desaturase* gene. J. Plant Biotechnol..

[252] Raj S., Saudagar P., Akhtar M.S., Swamy M.K. (2019). Plant cell culture as alternatives to produce secondary metabolites. Natural Bio-Active Compounds: Volume 3: Biotechnology, Bioengineering, and Molecular Approaches.

[253] Thirumurugan D., Cholarajan A., Raja S.S.S., Vijayakumar R., Vijayakumar R., Raja S.S.S. (2018). An Introductory Chapter. Secondary Metabolites. Secondary Metabolites—Sources and Applications.

[254] Yoon J.Y., Chung I.M., Thiruvengadam M. (2015). Evaluation of phenolic compounds, antioxidant and antimicrobial activities from transgenic hairy root cultures of gherkin (*Cucumis anguria* L.). S. Afr. J. Bot.

[255] Thiruvengadam M., Praveen N., Maria John K.M., Yang Y.S., Kim S.H., Chung I.M. (2014). Establishment of *Momordica charantia* hairy root cultures for the production of phenolic compounds and determination of their biological activities. Plant Cell Tissue Organ. Cult..

[256] Thiruvengadam M., Rekha K., Chung I.M. (2016). Induction of hairy roots by *Agrobacterium* rhizogenes-mediated transformation of spine gourd (*Momordica dioica* Roxb. ex. willd) for the assessment of phenolic compounds and biological activities. Sci. Hortic..

[257] Chung I.-M., Thiruvengadam M., Rekha K., Rajakumar G. (2016). Elicitation Enhanced the Production of Phenolic Compounds and Biological Activities in Hairy Root Cultures of Bitter melon (*Momordica charantia* L.). Braz. Arch. Biol. Technol..

[258] Swarna J., Ravindhran R. (2012). Agrobacterium rhizogenes–mediated hairy root induction of *Momordica charantia* Linn. and the detection of charantin, a potent hypoglycaemic agent in hairy roots. Res. J. Biotechnol..

[259] Rekha K., Thiruvengadam M., Sumita J. (2017). Secondary Metabolite Production in Transgenic Hairy Root Cultures of Cucurbits. Reference Series in Phytochemistry.

[260] Chang C.K., Chang K.S., Lin Y.C., Liu S.Y., Chen C.Y. (2005). Hairy root cultures of *Gynostemma pentaphyllum* (Thunb.) Makino: A promising approach for the production of gypenosides as an alternative of ginseng saponins. Biotechnol. Lett..

[261] Zhu F., Zhou Y.K., Ji Z.L., Chen X.R. (2018). The plant ribosome-inactivating proteins play important roles in defense against pathogens and insect pest attacks. Front. Plant Sci..

[262] Vivanco J.M., Flores H.E., Basra A.S. (2000). Control of root formation by plant growth regulators. Plant Growth Regulators in Agriculture and Horticulture: Their Role and Commercial Uses.

[263] Kamenosono M., Nishida H., Funatsu G. (1988). Agricultural and Biological Chemistry Isolation and Characterization of Two Luffins, Protein-biosynthesis Inhibitory Proteins from the Seeds of *Luffa cylindrica*. Agric. Biol. Chem..

[264] Di Toppi L.S., Gorini P., Properzi G., Barbieri L., Spane L. (1996). Production of ribosome-inactivating protein from hairy root cultures of *Luffa cylindrica* (L.) Roem. Plant Cell Rep..

[265] Poma A., Galeota K., Miranda M., Spanò L. (1997). A Ribosome-Inactivating Protein Principle from Hairy Roots and Seeds of *Luffa cylindrica* (L) Roem and its Cytotoxicity on Melanotic and Amelanotic Melanoma Cell Lines. Int. J. Pharmacogn..

[266] Mizukami H., Kondo T., Ogihara Y. (1997). Basic Proteins Produced by Hairy Root Cultures of *Trichosanthes Kiriliowii* var. *japonica*. Plant Biotechnol..

[267] Tarhan L., Kayali H.A., Urek R.O. (2007). In vitro antioxidant properties of *Cucurbita pepo* L. male and female flowers extracts. Plant Foods Hum. Nutr..

[268] Saha P., Kundu Sen S., Bala A., Mazumder U.K., Haldar P.K. (2011). Evaluation of anticancer activity of *Lagenaria siceraria* aerial parts. Int. J. Cancer Res..

[269] Erasto P., Mbwamb Z.H. (2009). Antioxidant activity and HPTLC profile of *Lagenaria siceraria* fruits. Tanzan. J. Health Res..

[270] Arora R., Kaur M., Gill N.S. (2011). Antioxidant activity and pharmacological evaluation of *Cucumis melo* var. *agrestis* methanolic seed extract. Res. J. Phytochem..

[271] Dhiman K., Gupta A., Sharma D.K., Gill N.S., Goyal A. (2012). A review on the medicinally important plants of the family cucurbitaceae. Asian J. Clin. Nutr..

[272] Kumar S., Kumar D., Jusha M., Saroha K., Singh N., Vashishta B. (2008). Antioxidant and free radical scavenging potential of *Citrullus colocynthis* (L.) Schrad. methanolic fruit extract. Acta Pharm..

[273] Rao A.V., Rao L.G. (2007). Carotenoids and human health. Pharmacol. Res..

[274] Alghasham A.A. (2013). Cucurbitacins: A Promising Target for Cancer Therapy. Int. J. Health Sci..

